# Alternative approaches to standard inpatient mental health care: development of a typology of service models

**DOI:** 10.1186/s13033-025-00669-7

**Published:** 2025-04-17

**Authors:** Jessica L. Griffiths, Helen Baldwin, Jerusaa Vasikaran, Ruby Jarvis, Ramya Pillutla, Katherine R. K. Saunders, Ruth E. Cooper, Una Foye, Luke Sheridan Rains, Molly Lusted-Challen, Phoebe Barnett, Geoff Brennan, Steven Pryjmachuk, Karen Newbigging, Jo Lomani, Rachel Rowan Olive, Lizzie Mitchell, Patrick Nyikavaranda, Chris Lynch, Karen Persaud, Brynmor Lloyd-Evans, Alan Simpson, Sonia Johnson

**Affiliations:** 1 NIHR Policy Research Unit in Mental Health (MHPRU), Department of Health Services and Population Research (HSPR), Institute of Psychiatry, Psychology & Neuroscience, King’s College London, London, UK; 2 NIHR Policy Research Unit in Mental Health (MHPRU), Division of Psychiatry, University College London, London, UK; 3 Independent Researcher, London, UK; 4 National Health Service (NHS) England, London, UK; 5 Centre for Outcomes Research and Effectiveness, Research Department of Clinical, Educational, & Health Psychology, University College London, London, UK; 6 Florence Nightingale Faculty of Nursing, Midwifery and Palliative Care, King’s College London, London, UK; 7 Department of Health Services and Population Research (HSPR), Institute of Psychiatry, Psychology & Neuroscience, King’s College London, London, UK; 8 School of Health Sciences, The University of Manchester, Manchester, UK; 9 School of Social Policy, University of Birmingham, Birmingham, UK; 10 NIHR Policy Research Unit in Mental Health Lived Experience Working Group, Division of Psychiatry, University College London, London, UK; 11 Department of Primary Care & Public Health, Brighton & Sussex Medical School, University of Sussex, Brighton, UK; 12 Camden and Islington NHS Foundation Trust, London, UK

**Keywords:** Inpatient mental health care alternatives, Typology development, Crisis care, Community care, Inpatient mental health care, Acute care

## Abstract

**Background:**

Inpatient mental health care is an integral part of the continuum of mental health care in many countries, though it can be associated with challenges, such as reliance on coercive practices, negative patient experiences, and limited therapeutic options. Given these issues, there is a growing interest in exploring alternative approaches for individuals experiencing a mental health crisis. This research aimed to identify models which offer an alternative to standard inpatient mental health care across all age groups, both nationally and internationally, and to develop a typology for these alternative models.

**Methods:**

A dual literature search and expert consultation research methodology was adopted to identify relevant models. Three typologies of models were developed according to age group and acuity, including: alternatives to standard acute inpatient services for adults; alternatives to longer-stay inpatient services for adults, including rehabilitation and forensic inpatient services; and alternatives to standard inpatient services for children and young people.

**Results:**

We identified an array of service models in each typology, some in community settings, some hospital-based and some working across settings. Models varied greatly in characteristics, extent of implementation and supporting evidence.

**Conclusions:**

Through this mapping exercise, we have developed three novel typologies of alternatives to standard inpatient care. A range of community-based, hospital-based and cross-setting approaches were identified. The identification of services providing inpatient care in a substantially different way to the standard suggests that some improvements could be provided within existing structures. Potential inequities in access to alternatives were identified for certain groups, such as people who are compulsorily detained, younger children, and young people transitioning between children’s and adults' services. These typologies can inform future description, evaluation and comparison of different service models. This research also yields some key considerations for the design, development and implementation of alternative mental health service models and service arrays.

**Supplementary Information:**

The online version contains supplementary material available at 10.1186/s13033-025-00669-7.

## Introduction

Inpatient mental health care is an important component of the mental health services system, both within the United Kingdom (UK) [1] and globally [2]. Inpatient services aim to offer intensive support and treatment of all modalities. Acute inpatient services admit people in crisis; longer-term wards are usually intended to focus on people with a high level of continuing need and/or risk. Stays are overnight and can result from either voluntary admission or involuntary admission in accordance with national law.

While inpatient care can play a critical role in supporting individuals with complex or high mental health support needs, there are ongoing challenges, including: high rates of coercive and restrictive practices, including involuntary hospitalisations [3, 4]; safety concerns, including risks of abuse [5, 6]; limited treatment choices [7]; over-reliance on medication [8, 9]; services not meeting the needs of minority ethnic groups [10–12]; and strained staff-service user relationships [13–15]. Service user critiques of hospitalisation highlight concerns about the potential for standard inpatient mental health care to violate service users’ human rights and freedoms [16].

Inpatient care is also costly; even though only 3% of people in England accessing mental health care in 2018/19 received inpatient mental health care, National Health Service (NHS) trusts in England still invest more in inpatient than community services [8]. Involuntary admissions have risen for several decades in England and some other high-income countries [17]. Thus, service user dissatisfaction and activism, concerns with justice and human rights, doubts about inpatient service effectiveness and cost pressures are among the drivers for the search for effective, cost-effective and acceptable alternatives to standard inpatient care.

In light of these challenges, the World Health Organisation has proposed substantial changes in global mental health systems to deliver care that is person-centred, rights-based, recovery-oriented, and addresses social determinants of health [18]. The quest for effective alternatives to standard hospital admission dates back to at least the mid-20th century, and has been a central concern in policy, service development and research for many decades, including both hospital-based approaches and community alternatives to both acute and long-term inpatient services [19, 20]. The goal is not necessarily to replace inpatient mental health care, as it is an important part of the care continuum and may be the model required or preferred by some individuals. However, offering alternatives could provide a more flexible range of support options within a stepped care approach, where a range of services are offered, from least to most intensive, based on individuals’ needs [21]. Currently, the availability of alternative models continues to vary greatly between and within countries, and innovative services are often small in scale, remain underequipped and underfunded [8].

Currently, there is no extended typology which identifies alternative approaches to inpatient mental health care internationally and across all settings and age groups. Such a typology has potential benefits to researchers, service planners and clinicians in increasing the extent to which alternatives may be systematically introduced in contexts where they are appropriate, evaluated and implemented. Including alternatives to inpatient care for children and young people (CYP) and in longer-term settings is also an advance on the focus of much previous literature focusing solely on alternatives to acute adult mental health care: very significant clinical, ethical and economic disadvantages have been identified both for hospital admissions for under 18s [22, 23] and for longer inpatient stays [24], so these are also valuable targets for the development, implementation of alternatives to standard inpatient care.

The National Institute for Health and Care Research (NIHR) Policy Research Unit in Mental Health (MHPRU) [25], a research team funded to deliver research evidence to inform mental health policy, has carried out this study following a request from policymakers in NHS England and the Department of Health and Social Care. The request arose from concerns about high rates of inpatient mental health service use, its quality, and consistent calls from service users for alternatives to standard inpatient mental health care. We sought to inform development and testing of inpatient and community alternatives to standard inpatient services and of integrated and comprehensive catchment area acute care systems by identifying, mapping out and categorising alternatives nationally and internationally, across all age groups. We aimed to develop three typologies of alternative approaches to standard inpatient mental health care according to: adult acute inpatient alternatives; alternatives to longer-stay inpatient services for adults (including rehabilitation and forensic inpatient services); and inpatient alternatives for CYP.

## Methods

### Study design

**Fig. 1:**
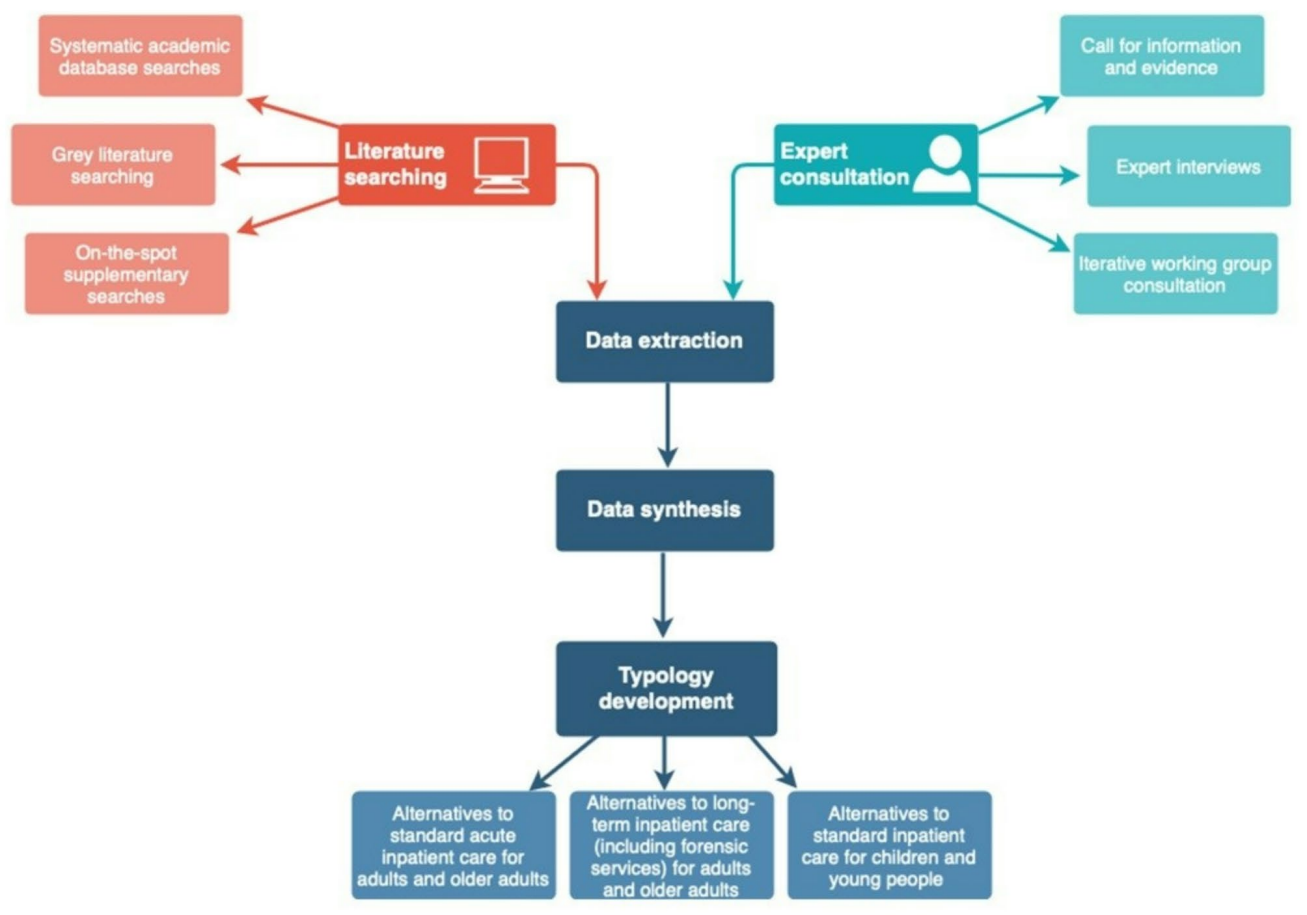
A flow diagram showing the research methodology used for typology development

This study’s methodology was informed by scoping review principles [26]. We used two simultaneous approaches for gathering data: literature scoping and a call for evidence from international experts (Fig. 1). Alongside this, an expert working group was established to inform the direction of the research and offer iterative consultation throughout. The working group comprised academics and researchers from the NIHR MHPRU with relevant experience in acute care, as well as clinical and professional experts (including psychiatrists, mental health nurses, clinical psychologists and social workers), lived experience researchers selected from the MHPRU’s Lived Experience Working Group (LEWG) who had experiences of different mental health difficulties and accessing different services, and policymaker representatives from NHS England. This working group included several well-established experts on inpatient mental health care and inpatient alternatives.

### Eligibility criteria

There is no universally agreed-upon definition of ‘standard inpatient mental health care’, and little discussion of its role, function, and design in the literature. What is considered ‘standard’ inpatient care varies across contexts and over time, with different perspectives on its purpose and function amongst patients, carers, and different types of professionals [27].

Bowers et al. (2009) suggest that admissions to acute inpatient mental health wards typically result due to severe acute mental health problems coupled with one or more of the following factors: high risk to self or others, refusal of treatment, life stressors, concerns about deterioration, failure to manage activities of daily living, and need for assessment [27]. In contrast, long-term and rehabilitation inpatient wards typically support people with complex and enduring mental health problems, and forensic wards support people in the criminal justice system or those deemed to be at high risk of harming others. Thresholds for inpatient admissions vary by ward types and local contexts, influenced by factors such as bed availability, social support, and other available services [27].

Some of the theorised key functions of inpatient mental health care include ensuring safety, providing assessment and psychiatric treatment, rehabilitation, meeting basic care needs, and physical health care. Support may also be provided for other issues, such as financial or housing problems [27]. Key components of inpatient care include: 24-hour staff presence, multidisciplinary team input, treatment provision, regular observation, containment, and tolerance of behaviour that would be unacceptable or unmanageable in the community. Inpatient admissions may also provide respite for patients, families and communities [27].

The absence of a universal definition for ‘standard’ inpatient mental health care makes it challenging to define ‘alternatives’ to it. For this study, we developed our own criteria for defining ‘alternatives to standard inpatient mental health care’, adapted from those used in a previous study by members of our team [28] and agreed upon amongst experts within our working group.

To be considered an alternative, each model had to aim to serve people who would otherwise be admitted to a standard acute psychiatric ward or receive longer-term inpatient psychiatric care (e.g., in secure or rehabilitation services), and meet **at least one** of the following criteria:

- Based outside of a hospital setting, including services that support people intensively at home, in day care settings or in community residential settings with the aim of reducing pressure on hospital services;
- Dedicated to delivering specialised care for a specific diagnostic or sociodemographic group, since standard inpatient services typically provide more generalised care;
- Have a fixed maximum length of stay, since standard inpatient care typically does not have one;
- Have implemented a specific therapeutic model, programme, or approach involving changes in the practice of more than one profession within a hospital or hospital alternative service, or that involves different types of workers in care;
- Have implemented a significant change in practice in the management of risk.

Models were included if they had the potential to either avoid inpatient admissions or shorten them by facilitating earlier discharge. Though most secondary community mental health models have a role in avoidance of hospitalisation, the focus here is on models that are more obviously intensive forms of support that could substitute for an inpatient admission, including during a period when someone is experiencing a mental health crisis.

We included models of care which fitted these criteria regardless of age range or geographical location, but, in the interests of feasibility, limited our scope through the exclusion of services specialising in care for perinatal populations, people with drug and alcohol problems, autistic people and people with intellectual disabilities, people living with dementia or other organic conditions, and solely prison-based services. These service types were also outside of the scope of policymakers’ request.

### Literature searching

#### Academic database searching

We conducted a broad initial search of relevant systematic and non-systematic reviews across three academic databases (PubMed, PsycINFO via Ovid and the Cochrane Central Register of Systematic Reviews) from the date of database initiation to 19th December 2022. Key words for inpatient alternatives and specific service models already identified by the working group were used (see Appendix A, Supplementary Material 1). We aimed to offer a broad scope of the literature at the review level and as such, the search strategy was thorough though not exhaustive.

Database records were exported to EndNote and screened for relevance by two researchers (HB, JG). Any uncertainties regarding a record’s eligibility were jointly discussed by JG and HB. If uncertainty remained, the multidisciplinary working group was consulted to reach a consensus on inclusion or exclusion. Given the interest in international models, non-English language documents were included and translated using Google Translate. Extracted information was checked by someone with a knowledge of the language.

#### Grey literature searching

The academic literature searching was supplemented by grey literature searching using Google and nine other grey literature databases (see Appendix B, Supplementary Material 1) identified by the expert working group. Grey literature searches were conducted by a member of the research team (RP) between 16th December 2022 and 1st February 2023. There were no restrictions on the type of sources that could be included from grey literature searching. Any uncertainties regarding the eligibility of grey literature sources were discussed between RP, JG, and HB, and, when necessary, with the wider multidisciplinary working group.

#### Supplementary searches

Supplemental searches of academic databases and grey literature were conducted for specific service models where there were gaps or insufficient detail in the accrued resources. These gaps were identified by reviewing the data extraction form for each model (see Supplementary Material 2). There were no restrictions on the types of sources that could be included from supplementary searches.

### Expert consultation

The literature scoping workstream was supplemented with expert consultation. The expert working group identified key international experts (see Acknowledgements) to be contacted with a call for information (see Appendix C, Supplementary Material 1). 120 experts (including health care professionals [*n* = 26], academics [*n* = 32], clinical academics [*n* = 35], charity workers [*n* = 13], experts by experience [*n* = 6], and policymakers [*n* = 8]) were asked via email to provide information relating to relevant models, any literature associated with these models, and recommendations for other experts to contact. Using this snowballing approach, a further 18 experts were contacted, resulting in a total of 138 experts being individually contacted. The call for information was also distributed to 26 specialist clinical (*n* = 3), academic (*n* = 4), charitable/not-for profit (*n* = 7), lived experience (*n* = 3), and mixed (*n* = 9) networks and organisations operating internationally and identified by the expert working group.

There were no specific criteria that experts needed to meet in order to be contacted with our call for information. All literature and alternative models recommended by experts were screened for relevancy by two researchers (JG, HB). Any uncertainties regarding eligibility were discussed between JG and HB and if needed, with the multidisciplinary working group, to reach a consensus on whether to include or exclude them. Data were extracted for those that met our eligibility criteria. There were no restrictions on the types of sources that could be included from expert consultations.

The call for information was delivered to the identified experts via email, and responses were received either via video call or email. A semi-structured interview guide was developed (JG, HB) for this study to facilitate discussions with experts via video (see Appendix D, Supplementary Material 1). Monetary compensation (£50) was provided to contributors from the voluntary/third sector who provided information via video expert consultation. Interviews with experts were conducted jointly by two researchers (JG, HB), who made notes during the interviews to capture the discussions. Given that the primary aim of these expert consultations was to identify alternative models, no formal qualitative analysis of these notes was performed, but the researchers followed-up by screening all literature recommendations made and conducting supplementary searches to investigate any alternative models mentioned in the interviews.

### Data extraction and synthesis

All relevant models and services identified from the literature scoping and expert consultation were extracted in Microsoft Excel by one of the research team (HB, JG, RJ, JV, KS, RC, RP). This data extraction form was designed following consultation with the expert working group and piloted on a set of studies initially in case modifications were needed. A range of key model descriptor variables were extracted, including: setting; funding; location; brief description of the model; date of establishment; target population; occupational roles of staff involved in the model; typical duration of support; access routes; whether the service can accept compulsorily detained individuals; and the details of any associated literature.

Where identified, additional information was also extracted regarding quantitative service use outcomes, including inpatient admissions/readmissions; number of inpatient bed days; length of stay; and satisfaction with care (see Supplementary Material 2). It should be noted that the outcome evidence presented was not subjected to quality appraisal and is not exhaustive.

Extracted models were then synthesised into three separate typologies where they were broadly categorised according to their settings, approaches and target populations. A decision was made by the working group early on in the typology development process to stratify the typologies by age and target population, rather than presenting a single comprehensive typology. This was for ease of interpretation, given the large number of models included, and to facilitate comparisons across the different populations.

The typology categories were iteratively developed using inductive and deductive methods. Feedback on the typologies was sought from the working group during regular working group meetings. Where knowledge gaps in the expert working group were identified, additional external experts were consulted. Expert feedback was used to refine the typologies until a consensus was reached on their content and format among all working group members.

### Ethics approval and consent to participate

Minimal risk ethics was obtained from the King’s College London ethics committee (Approval Number: MRA-22/23-34963). In accordance with ethical approval, an information sheet and consent form were attached to all call for information emails to experts. The information sheet detailed the study’s purpose and methods. The consent form explicitly explained that providing information to the research team would be considered confirmation of consent to participate, unless participants explicitly stated otherwise. This process ensured that all participants provided informed consent regarding their involvement in this study.

## Results

### Literature searching and expert consultation

Database searches returned 4,253 studies and a total of 78 experts responded to our call for information, from a range of countries. Their professional backgrounds included: academics, health care professionals, clinical academics, experts by experience, policymakers, charity workers and service leaders (see Appendix E, Supplementary Material 1 for more detailed breakdown). Screening of database search results and literature recommendations from experts for eligibility, combined with supplementary searches, resulted in the inclusion of 435 relevant sources. These sources ranged from published peer-reviewed literature to policy documents, websites, books and videos.

### Typology mapping

Identified models were categorised in three typology maps: (i) alternatives to adult standard acute inpatient care (see Supplementary Material 3), (ii) alternatives to adult standard long-term inpatient care (including rehabilitation and forensic inpatient care) (see Supplementary Material 4), and (iii) alternatives to standard inpatient care for CYP (see Supplementary Material 5). Table 1 provides a high-level overview of service model comparisons across the three typologies.

**Table 1:** A high-level summary and comparison of the models of care across each of the typologies

		Adult acute inpatient alternatives	Adult long-term rehabilitation and forensic inpatient alternatives	Children and young people inpatient alternatives
**Community-based alternatives**				
Crisis houses	Peer-led crisis houses	x		
	Clinical crisis houses	x		x
	Non-clinical crisis houses	x		
	Specialist crisis houses	x		x
Intensive residential services	Residential rehabilitation services		x	x
	Intensive supported housing models		x	x
Secure accommodation	Secure children’s homes			x
	Secure training centres			x
	Residences for the execution of security measures		x	
Family placement schemes	Family sponsor homes	x		
	Shared lives	x	x	x
	Therapeutic foster care			x
	Geel family foster care model		x	x
	Healing homes		x	
Acute day services	General acute day units	x		x
	Specialist acute day units	x		x
	Enhanced acute day treatment	x		
Home based crisis services	Crisis resolution and home treatment teams	x		x
	Other intensive home treatment services	x		x
	Intensive home treatment with optional brief inpatient admission			x
	Homebuilders model			x
Emergency service linked models	Police and/or ambulance street triage	x		x
	Clinician-only mobile crisis unit	x		x
Drop-in crisis services	Crisis assessment services	x		x
	Lifeguard pharmacies	x		
	Mental health crisis hubs	x		x
	Crisis cafes	x		x
Discharge transition support	Transition to recovery program	x		
	Supported discharge service			x
	Hot-BITS			x
	Peer-Bridger	x	x	
Outpatient-based crisis services	Whole of service stepped care approach	x		x
	Behavioural health crisis care clinic			x
General community services with crisis function	Enhanced case management	x	x	x
	Enhanced community mental health teams	x		
	Early intervention models	x		x
	Adolescent forensic community services			x
	Intensive intervention and risk management services		x	
Specialist community mental health rehabilitation teams	Specialist community mental health rehabilitation teams		x	
**Hospital-based alternatives**				
Models involving general hospital medical care	Enhanced psychiatric liaison services	x		x
	Time limited admission to general medical wards with specialist community eating disorder team input	x		x
Brief stay crisis units	23-hour crisis stabilisation units	x		x
	Behavioural assessment units	x		x
	Psychiatric emergency service centres	x		x
	Psychiatric observation units	x		
	Psychiatric decision units	x		
	Emergency psychiatric assessment, treatment and healing units (EmPATH units)	x		
Inpatient psychiatric services	Services with a specific therapeutic model	x	x	x
	Inpatient wards for specific groups	x	x	x
	Short-stay acute inpatient wards	x		x
	Ter beschikking stelling (TBS)		x	
**Cross-setting approaches**				
Open dialogue	Open dialogue	x		x
	Peer supported open dialogue	x		
Therapeutic communities	Therapeutic communities	x	x	x
	Democratic therapeutic communities	x	x	x
Other cross-setting approaches	Need-adapted treatment	x		x
	Specialist consultancy	x	x	
	The sanctuary model	x	x	x
	Enabling environments	x	x	x
	Wraparound with intensive services	x		x
	Trieste	x	x	x
	Multi-systemic therapy			x
	Psychologically informed planned environments		x	
	Psychologically enhanced resettlement services		x	
	Offender personality disorder treatment services		x	

In total, we identified 65 distinct alternative models. Of these, 20 provided support to adults only, 10 to CYP only, and 35 had evidence of use with both adults and CYP. This highlights the considerable overlap between the models featuring in the adult and CYP typologies.

Among the 55 models providing support to adults, 33 were alternatives to standard acute inpatient care, 11 were alternatives to long-term inpatient care (including rehabilitation or forensic services), and 11 offered an alternative to both acute and long-term standard adult inpatient care. Six of the alternative models for adults represent components of the Offender Personality Disorder pathway in the UK, which operates exclusively in forensic contexts. This aims to offer a pathway of psychologically informed services for offenders with likely “severe personality disorder” [29].

Models in each typology were broadly categorised into community-based alternatives, hospital-based alternatives or cross-setting approaches. Community-based approaches encompass models which are home-based or located within another community setting, rather than in a traditional hospital setting. Hospital-based models are offered within a hospital setting, including inpatient wards operating in a substantially different way to standard inpatient care according to our criteria. Finally, cross-setting approaches are broader frameworks or philosophies that can be implemented across different types of settings whilst maintaining their core principles and values and often take a systemic perspective. Across all three typologies, 39 alternative models were community-based, 12 were hospital-based, and 14 were cross-setting approaches.

Examples of community-based alternatives include community residential models (such as crisis houses and secure accommodation), family placement schemes, acute day services, home-based crisis care (such as crisis resolution and home treatment teams), emergency service-linked models, drop-in crisis care models (such as crisis cafes), outpatient-based crisis services, discharge transition services which aid with return to the community, and general community services which integrate a crisis response component (such as enhanced community mental health teams and early intervention in psychosis services).

Examples of hospital-based alternatives include models involving general hospital medical care (such as extended psychiatric liaison services), brief-stay crisis units (such as psychiatric decision units), and inpatient psychiatric services which operate in a substantially different way to standard inpatient care according to our criteria, such as inpatient services implementing a specific therapeutic model (such as Safewards), wards specialised for different groups (for example, Deaf people or people with particular psychiatric diagnoses) or short-stay acute inpatient wards.

Examples of cross-setting approaches include ‘therapeutic communities’, ‘Open Dialogue’, ‘multisystemic therapy’, ‘enabling environments’, and Trieste, a whole-system approach.

The sectors delivering these models varied: 33 were provided solely by the public sector, three solely by the voluntary or third sector, three solely by the private sector, and 24 were delivered by a combination of sectors. For two models, the providing sector could not be determined.

Across all three typologies, six models were identified as peer-led or as having peer-led variants, including peer-led crisis houses, peer-led crisis cafes, and ‘peer-supported Open Dialogue’. Five models aimed to provide tailored care to specific sociodemographic groups, including women, veterans, minoritised ethnic groups, Deaf people, or people experiencing homelessness or housing instability. Additionally, some models aimed to provide specialised care for certain clinical groups, including those experiencing psychosis, suicidality, eating disorders, or with a diagnosis of “personality disorder”.

A more in-depth description of all the models, their accompanying references, and identified quantitative evidence (relating to outcomes including admission/readmission rates, inpatient bed days, length of stay and satisfaction with care) is provided in Supplementary Material2, Supplementary Material6 and Supplementary Material7.

## Discussion

### Key findings

The typologies we have presented show that a wide variety of approaches exist internationally which may act as an alternative to standard acute inpatient mental health care for adults and CYP, and to standard long-term and forensic inpatient care across a range of settings.

While we identified a range of community-based approaches, including some peer-led approaches, we also noted services which provide inpatient care in a substantially different way to the standard, including time-limited approaches, inpatient services with a particular therapeutic model (including trauma-informed approaches), and service models which aimed to reduce coercion and restrictive practice. Inpatient care is an important component of the mental health system, and these non-standard inpatient service models demonstrate that some improvements to care can be provided within existing inpatient structures. However, the fundamental needs of services, such as adequate resourcing and staffing, and workforce development, must be met if good quality crisis or rehabilitation care is to be provided, and we cannot assume that these innovations eliminate coercive or otherwise harmful elements of hospitalisation.

We also observed that exclusion criteria for the alternative models seemed to vary between individual services (see Supplementary Material 7) and were often not stated in the literature. However, where this information was available, compulsory detention was a common exclusion criterion. Other exclusion criteria for some services included: people assessed as presenting with high risk to themselves or others, people with substance abuse difficulties and people with intellectual disabilities. This inequitable access to alternative models could have several disadvantages for these individuals, including limiting their choice of care, prolonging institutionalisation in more restrictive settings, reinforcing feelings of stigmatisation and disempowerment, and exacerbating distress. Furthermore, given that these are common comorbidities in mental health populations, this poses the question as to whether the identified models truly act as alternatives to inpatient care, if substantial numbers of those receiving inpatient care are excluded from accessing them. This is compounded by further observations that certain groups were not fully considered in existing service provisions. For example, there appeared to be relatively fewer models providing support to younger CYP, and CYP transitioning from children’s services to adult services. In line with previous literature [30, 31], these observations reiterate that key gaps exist in service provisions for these populations. Mental health care services should not necessarily be considered as a one-size-fits-all approach– more granular consideration of sociodemographic and diagnostic factors in service delivery may help to improve the quality of care [32]. Taken together, this necessitates the implementation of alternative models which can serve these groups, including services which can intervene before the point of detention for people at high risk.

We also identified a range of voluntary sector led models (such as ‘non-clinical crisis houses’, and ‘crisis cafés’) that make an important contribution to supporting individuals in crisis by providing an immediate response, contributing to prevention and recovery, and through the provision of a social rather than clinical approach (see Supplementary Material 2 and Supplementary Material 7). Evidence suggests that voluntary sector organisations are attractive and acceptable to people in crisis and can be more attractive to minoritised groups than statutory services [33]. However, there are still some documented issues with geographical variability in service availability and inequalities in access for certain minoritised groups [33]. Indeed, it is often voluntary and third sector organisations that address these gaps in provision to offer more culturally appropriate care to marginalised and minoritised groups. However, voluntary and third sector organisations often have short-term, unpredictable funding compared to statutory services, limiting their capacity to conduct rigorous research needed for larger-scale implementation. This may contribute to their underrepresentation in the existing literature base. Encouraging collaboration between the voluntary sector and health and social care researchers could help to address this gap. Further quantitative and qualitative research is needed to evaluate different voluntary sector models, including their outcomes, their impact on promoting equality and their partnerships with public sector services [33].

### Strengths and limitations

In this research, a comprehensive approach was taken to identify various inpatient alternatives. The search process involved reviewing literature and consulting experts to identify a wide range of alternative models. The inclusion criteria were broad, allowing for the inclusion of alternatives from any country, time period, and for people of any age. Whilst systematic academic database searches were limited to reviews to ensure manageable screening, there were no restrictions on the type of sources that could be included from grey literature or supplementary searches, or expert consultations. Models were also included irrespective of the level of evidence available for them. This ensured a comprehensive scoping of alternatives. Furthermore, key stakeholders were consulted throughout the project to ensure the real-world applicability of the resulting typology.

Indeed, the development of international typologies of alternative service models offers a clear framework for understanding and categorising different types of mental health support. This can help to improve our understanding of international mental health care provision and, in turn, inform service planning, delivery and policymaking. These typologies can also help to identify gaps in local service provision, and to drive research and evaluation efforts for models which have received less funding and attention. These typologies may therefore be helpful for researchers, mental health professionals, policymakers and service users alike.

However, there are also limitations to consider in both the research process and the interpretability of our findings. It should be acknowledged our scoping exercise aimed to be broad and rapid, and its aim was to identify and describe existing models, not to evaluate their effectiveness. Though key information and literature associated with each model is provided, full systematic reviews which follow PRISMA guidelines [34] are required for a comprehensive description of each model and its associated evidence to better inform funding decisions and resource allocations.

Our working group included well-established experts in inpatient mental health care and inpatient alternatives, and we conducted extensive international consultations with a diverse range of experts, including academics, health care professionals, clinical academics, experts by experience, policymakers, charity workers and service leaders. This collaborative approach helped to ensure a comprehensive identification of inpatient alternatives. However, we acknowledge that despite this, there may be some models that were missed. The majority of experts who responded to our call for information and working group members were based in England, so models from other parts of the UK and international models may not have been adequately captured. Additionally, the England-centric nature of the working group may have also influenced our definition of what is considered ‘standard’ within practice. Another limitation is that the typologies do not capture models that fall outside of the scope of this study, including perinatal services, addiction services, services specifically for autistic people or people with intellectual disabilities, neurorehabilitation services, services for people living with dementia, and solely prison-based services.

There is no standardised definition of ‘standard inpatient mental health care’, as it varies across different contexts and over time. Consequently, what can be considered an ‘alternative’ to standard inpatient care is subjective. For this study, we developed our own criteria for ‘alternatives’ to standard inpatient care based on previous research on inpatient alternatives (e.g., Johnson et al., 2009 [28]) and consensus with academic, lived experience, and clinical experts with specialist knowledge of inpatient mental health services and alternatives. Had other criteria been applied, different models may have been included or excluded in the typologies.

Furthermore, the typology development involved the categorisation of complex and diverse models into distinct groups. This can oversimplify how these models operate in practice and the heterogeneity within each model. In practice, the boundaries between different models may be more blurred. Similarly, there may also be variability in outcomes depending on how models are implemented and the contexts in which they operate. Whilst presenting the three separate typologies offered greater clarity for ease of interpretation, there was a large degree of overlap between both the CYP and adult models of care, and the acute and long-term models of care. Therefore, the distinctions between the different typologies can also be blurred. In future, alternative approaches to typology development could be taken– for example, with different scope or categorising models on the basis of different features (e.g., function versus form). Mental health service delivery is constantly changing, and so new models and approaches are likely to emerge over time; this typology provides a strong foundation which future research can build upon.

The focus of our typology on crisis provision constrains its ability to adopt a comprehensive system-wide approach that considers broader care pathways. It is important to consider the conceptualisation of mental health crises as ‘biographical disruptions’– intense and extreme experiences which disrupt everyday life and potentially have far-reaching consequences– rather than episodes requiring an urgent response [33]. This perspective highlights the importance of providing support not only during crises, but also before and after. Inpatient mental health services are vital for some. However, offering alternative options within a stepped care approach, where individuals can first access lower-intensity support such as drop-in community-based services or at-home care before progressing to higher-intensity interventions, could enable access to more flexible and tailored support. It could also be argued that effective mental health care at earlier stages, such as primary and preventative care, has the potential to prevent crises from occurring. Additionally, action to address systemic contributors to distress (e.g., social and economic inequalities, trauma, discrimination and marginalisation, limited community resources and social support) could help to promote wellbeing and prevent crises. This includes, for example, racism and poverty, which intersect with other inequalities to exacerbate distress and limit access to care. Addressing such factors could involve policy changes, social reforms, advocacy and community empowerment. This highlights the importance of taking action to prevent crises and reduce the social determinants of mental ill health, not just focusing on crisis care provision.

### Implications for research, policy and practice

It is apparent from this work, and existing literature, that crisis alternatives have proliferated in recent decades and crisis systems have typically become more complex [35]. However, there is a limited evidence base to inform service planners’ decision-making. This study has focused on mapping out three international typologies of service models which may offer an alternative to standard acute and long-term inpatient mental health care (including in inpatient rehabilitation and forensic settings) for adults and CYP. These typologies encompass community-based, hospital-based, and cross-setting approaches, including some models that offer innovative approaches to providing care within inpatient mental health services themselves. Whilst inpatient care forms an integral component of the continuum of mental health care, the availability of alternative options within a stepped care approach can ensure more flexible and tailored support. Indeed, our findings demonstrate that a range of alternatives are already available and are currently being successfully implemented.

Service users, carers, mental health professionals, charities and advocacy groups, and some government agencies and health authorities have called for improvements to inpatient care and alternatives to inpatient admissions [1, 33, 36–41]. Furthermore, concerns have been raised that involuntary treatment in inpatient mental health services may infringe upon individuals’ human rights [42]. This underscores the need for further investment and research into alternatives to standard inpatient health care. Our typologies can help service planners and commissioners recognise and consider the whole range of options when attempting to decide which crisis system improvements to prioritise and invest in, as well as guiding researchers in future attempts to explore the critical ingredients of crisis care systems.

Whilst we did not systematically assess the availability of the included models in this study, existing research suggests that the availability of many crisis services varies substantially geographically [35]. A key constraint about the usefulness of models is the degree to which it has been feasible to implement them. Future research could further investigate the feasibility of implementing models in different contexts, including exploring barriers and facilitators to effective implementation. However, geographical variation in availability may also result from services being commissioned to meet specific local needs. Recent research reports few associations between any particular community crisis model and system-level outcomes– perhaps reflecting that the quality of care is most important [43]. Some efforts have been made to define standards, key components and best practices of effective crisis services and pathways [44, 45], which could help to improve the quality of crisis care. For example, research has shown that increasing model fidelity in crisis resolution and home treatment teams leads to reductions in inpatient admissions [46]. Future research could continue to develop and refine these best practice standards, investigate methods to enhance fidelity to them, and examine the impact of this upon outcomes and experiences of care.

Future research efforts should continue to evaluate these alternative models to better understand their effectiveness in practice, factors influencing their outcomes, and how they can be most effectively integrated into a crisis response system, and what works best for whom, when and how - prioritising models with an established and/or promising evidence base. It is important to involve other sectors, particularly the voluntary and third sector, in this research to ensure their contributions are appropriately represented. Crucially, a focus on co-produced research is needed with people with lived experience of mental health difficulties, their families, clinicians, staff from voluntary and third sector organisations, policymakers, and commissioners to ensure specific service models in specific areas address the priorities of these key stakeholder groups.

## Conclusions

Through literature scoping and expert consultation, we developed three novel typologies: alternatives to standard acute inpatient mental health services for adults, alternatives to longer-stay inpatient services for adults (including rehabilitation and forensic inpatient services), and alternatives to standard inpatient services for CYP. These included a range of community-based, hospital-based and cross-setting approaches. Potential inequities in access were identified for certain groups, such as those compulsorily detained, younger CYP, and those transitioning between children’s and adults’ services. Whilst inpatient mental health services remain an integral part of the mental health care continuum, these typologies can inform the description, evaluation, and comparison of different service models, offering key insights for the design, development and implementation of alternative mental health service models and crisis systems.

### Lived experience commentary, written by ROO, LM and KP

As compulsory hospital admissions rise in the UK, providing alternatives is vital. However, diverting someone’s care pathway around the most intense crisis point is not the only, or necessarily the best, way to reduce admissions– many admissions result from unmet needs elsewhere. It is vital to consider the whole ecosystem of care, including social safety nets, social work, and social care, which have been drastically eroded over the last decade and a half by austerity and a global pandemic. We are tired of continually suffering, and watching others suffer, avoidable crises triggered by punitive benefits reviews, workplace discrimination, housing problems, and/or lack of compassionate community services. For carers, watching loved ones be traumatised by repeated - sometimes punitive - admissions leads to hopelessness and relationship breakdowns. This is compounded when carers are not identified or included in care by those arranging admissions.

Disparities in detention rates suggest racialised groups, especially Black patients, cannot access alternatives to hospital equally or at the right time. Nor are racialised groups as likely as White patients to receive therapy [47–49] despite the World Health Organisation emphasising everyone’s rights to choice and meaningful support. Bearing this in mind, institutional racism will continually influence access to admission alternatives unless it is actively and consciously countered with culturally sensitive commissioning, valuing service user and carer voices. While it is tempting to seek scalable models to roll out nationwide, we also need local flexibility to work with local communities’ needs. This is a constant tension in policy work: the flipside of flexibility is a postcode lottery. It has been eye-opening to read about alternatives unavailable in our areas and disappointing to realise how much some groups are missing.

For the services that do currently exist, accessibility is an issue. Many people who need these services will not know they exist, and many services are ‘gatekept’, requiring a professional referral in order to receive support - but if you are not under a professional’s care, or the professional does not think you are suitable, then you won’t be able to access the service. Finally, a system at breaking point cannot deliver admission alternatives as intended. We have seen cracks in the UK system open wide enough to swallow us and our loved ones whole: people waiting weeks in a 24-hour assessment unit; living in “supported” housing offering little meaningful support; unable to work with crisis teams failing to provide basic consistency. We are left wondering how new admission alternatives can possibly be implemented in this climate; how such services can provide accountability to users and carers; and worried that overstretched providers might not resource them sufficiently to be safe.

## Electronic supplementary material

Below is the link to the electronic supplementary material.

Supplementary Material 1

Supplementary Material 2

Supplementary Material 3

Supplementary Material 4

Supplementary Material 5

Supplementary Material 6

Supplementary Material 7

## Data Availability

The Excel dataset generated via expert consultation and literature searching is available within this manuscript’s supplementary files. Unpublished data and identifiable information from expert responses have not been made publicly available to protect participants’ confidentiality.
