# Supplementary figures and images for: Alternative approaches to standard inpatient mental health care: development of a typology of service models
Source: Int J Ment Health Syst. 2025 Apr 17;19:13. doi: 10.1186/s13033-025-00669-7 (PMC12007381; doi:10.1186/s13033-025-00669-7)

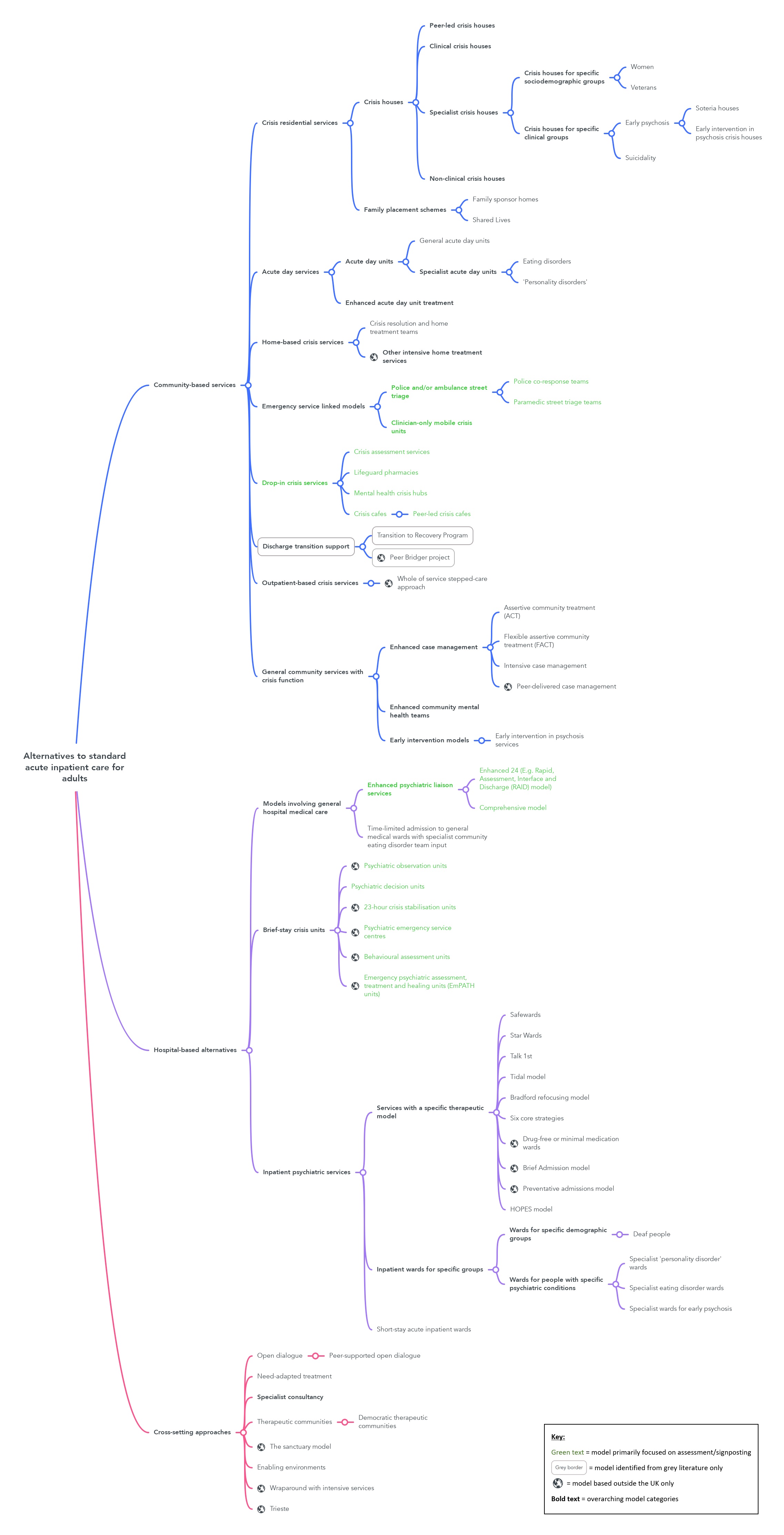

Supplement: Supplementary file 3 — Supplementary Material 3 [file 13033_2025_669_MOESM3_ESM.jpg]

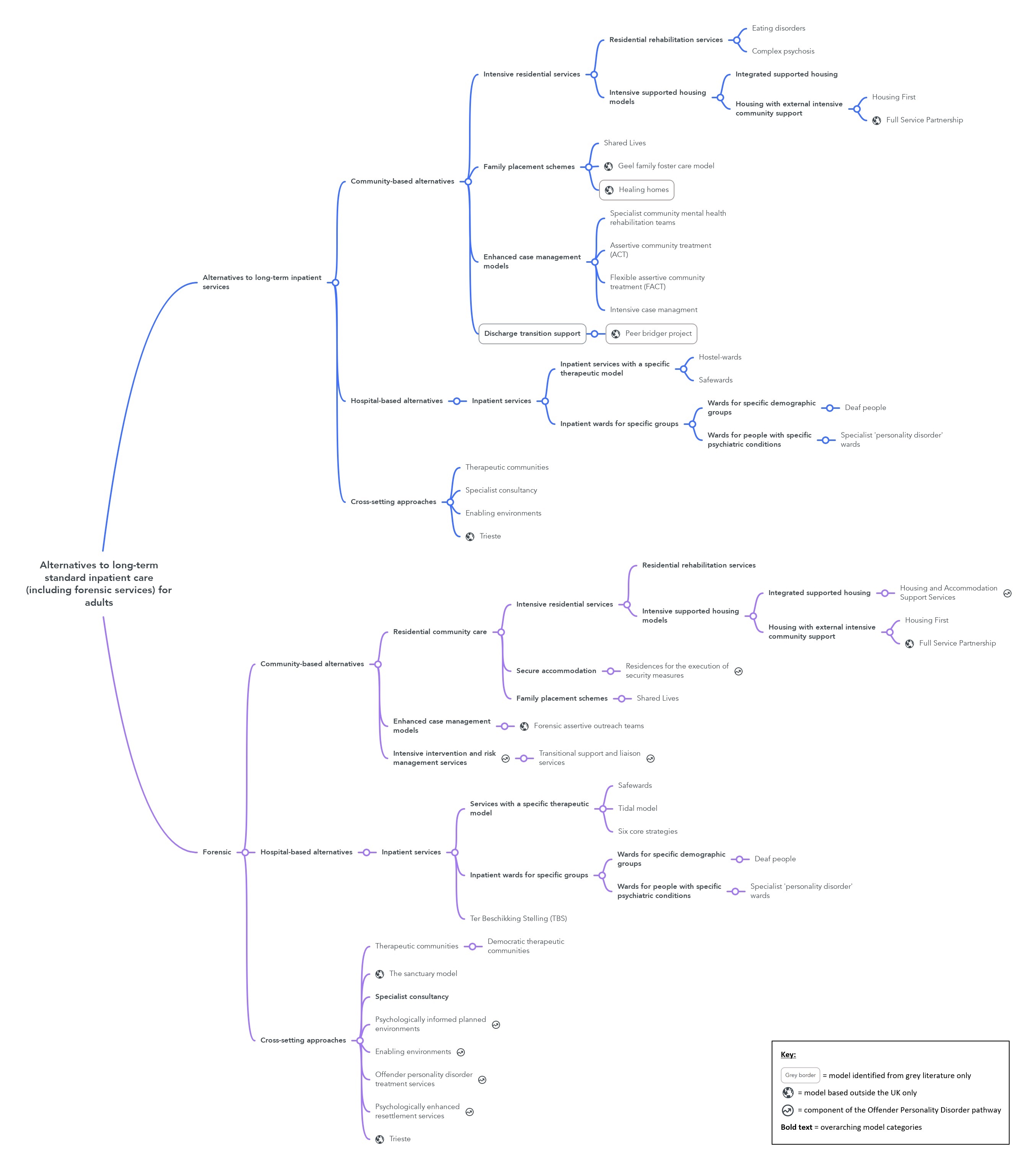

Supplement: Supplementary file 4 — Supplementary Material 4 [file 13033_2025_669_MOESM4_ESM.jpg]

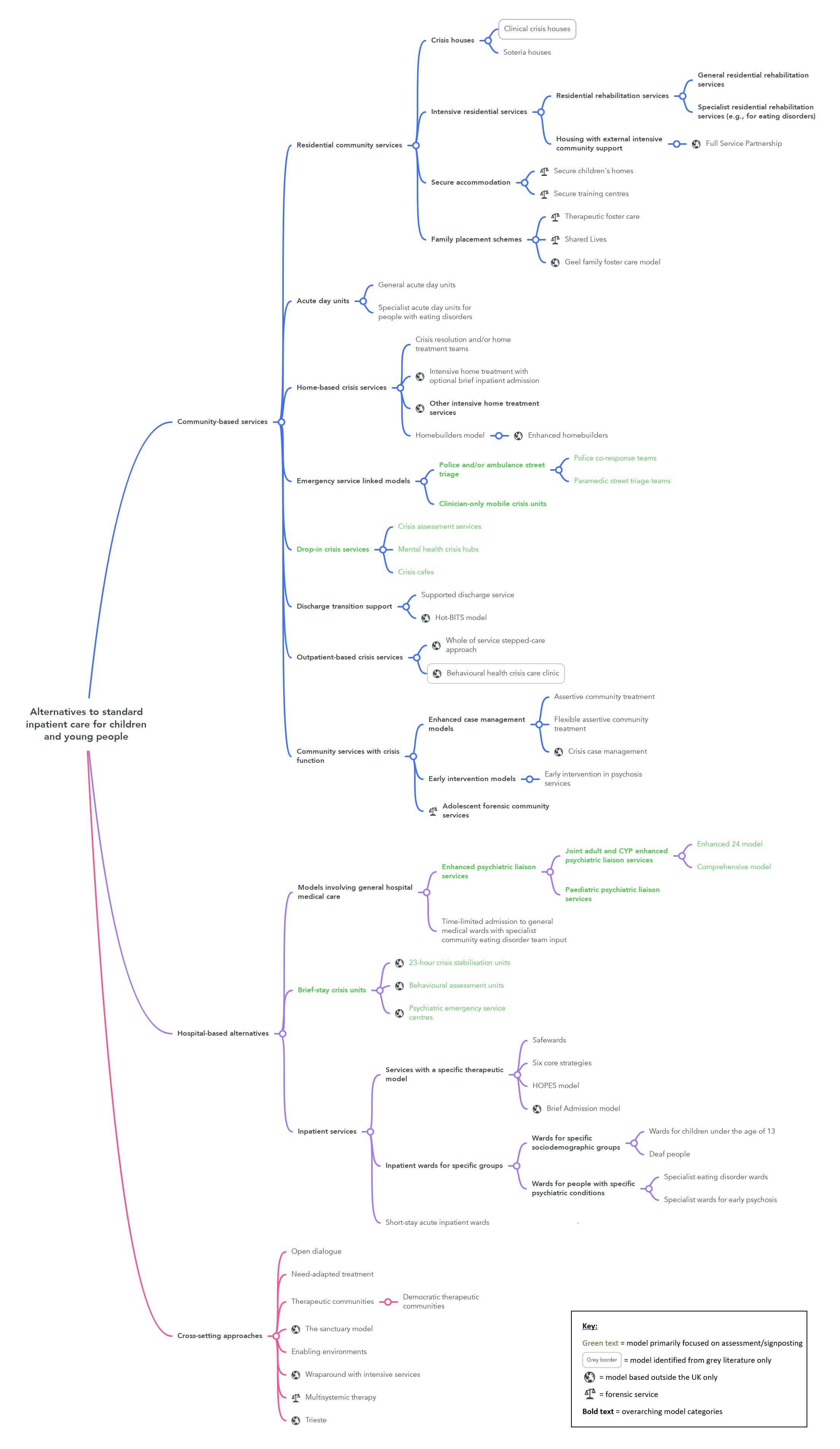

Supplement: Supplementary file 5 — Supplementary Material 5 [file 13033_2025_669_MOESM5_ESM.jpg]
