# Supplementary material for: Alternative approaches to standard inpatient mental health care: development of a typology of service models
Source: Int J Ment Health Syst. 2025 Apr 17;19:13. doi: 10.1186/s13033-025-00669-7 (PMC12007381; doi:10.1186/s13033-025-00669-7)
Supplement: Supplementary file 6 — Supplementary Material 6 [file 13033_2025_669_MOESM6_ESM.docx]

# Supplementary Material 6

This document provides more detailed descriptions of the alternative models featured within each of the three typologies, broken down into the following sections:

1. Alternatives to adult standard acute inpatient care (see Supplementary Material 3)
2. Adult long-term inpatient care alternatives (see Supplementary Material 4)
3. Adult long-term inpatient care alternatives: forensic (see Supplementary Material 4)
4. CYP alternatives: acute, long-term and forensic (see Supplementary Material 5)

## 1. Adult acute inpatient alternatives

This section describes services intended to support adults in a mental health crisis that may otherwise result in admission to a standard acute inpatient ward (see Supplementary Material 3). Models aiming to shorten acute inpatient ward stays by facilitating earlier discharge were also included. Further information, and associated outcome evidence identified, for each model is provided in Supplementary Material 2 and Supplementary Material 7.

### 1.1. Community-based alternatives

Crisis residential services

‘Crisis residential services’ provide temporary accommodation and support to people in crisis, typically for periods ranging from a few days to a few weeks. They include ‘Crisis houses’, which vary in approach, ranging from clinical to non-clinical. ‘Non-clinical crisis houses’ are usually run by voluntary sector organisations; they have fewer clinically qualified staff and employ approaches more clearly distinct from those in standard acute settings than ‘Clinical crisis houses’ (1). Crisis houses can be peer-led and can cater to specific sociodemographic groups, such as women or veterans, or specific clinical groups, such as people experiencing suicidality or early psychosis (e.g., Soteria houses) (1–5). The original Soteria model involves non-medicalised, community-based, residential support for people experiencing acute psychotic episodes in a home-like environment. It emphasises personal relationships, minimal hierarchies between staff and service users, minimal use of medication and restraint, and holistic care (1,6).

This category also includes ‘Family placement schemes’ (including ‘Family sponsor homes’ and ‘Shared Lives’) which place individuals with volunteer host families or carers at times of crisis. ‘Family sponsor homes’ support people for up to four weeks (7) whereas ‘Shared Lives’ can offer long-term, short-term or day-support placements (8).

Acute day units

‘Acute day units’ offer intensive, non-residential treatment and support for people facing acute mental health issues, offering an intermediate level of care between inpatient hospitalisation and outpatient services. ‘Enhanced acute day unit treatment’ models augment the standard model in some way, for example by providing additional respite or outreach services, crisis beds, or extended hours programmes (9). Acute day units tend to follow a structured schedule, normally operating during regular business hours (10). The intensity of programmes varies, and they tend to include a combination of individual therapy, group therapy, medication management, and support with practical issues (e.g., housing, welfare benefits, legal issues) and physical health (10). Some acute day units are designed for specific groups, such as people with diagnoses of eating disorders or “personality disorders” (11). ADUs may aim to avoid inpatient admissions or shorten inpatient stays (10). They can be part of step-down programmes, where people engage in brief intensive acute day unit treatment followed by a period of longer-term therapy (12,13).

Home-based crisis services

‘Home-based crisis models’ include ‘Crisis Resolution and Home Treatment Teams’ (CRHTTs) that provide intensive support and treatment to people in crisis, operate 24/7 and offer a rapid response (14). They often act as a gatekeeper for inpatient admissions (14). They are intended to avoid inpatient admissions and facilitate earlier discharge from hospital. They are staffed by multidisciplinary teams which can provide medication management, safety planning and psychological and social interventions (15). CRHTTs can provide both assessment and treatment, or these functions can be performed by separate teams. ‘Other intensive home treatment services’ can provide similar support, which may be staffed by psychiatrists, nurses, psychologists, social workers, therapists or peer workers (16–21).

Emergency service linked models

‘Paramedic and/or ambulance street triage teams’ are specialised units staffed by mental health clinicians and emergency service staff. Sometimes peer workers are also integrated into these teams. They provide immediate assessment and intervention to people in crisis who come into contact with law enforcement or emergency services in community settings (7,18,22). They aim to divert people from unnecessary arrest or incarceration. These teams can be ‘Police co-response teams’ or ‘Paramedic street triage teams’, and their formats can vary, with some providing in-person support from a mental health clinician during calls, and others where clinicians provide telephone advice only (7,18). ‘Clinician-only mobile crisis units’ perform similar functions but are only staffed by mental and physical health clinicians.

Drop-in crisis services

‘Drop-in crisis services’ provide immediate support to people experiencing a mental health crisis without the need for an appointment, as well as signposting and triage to further crisis support for those requiring urgent care. Some operate during extended hours, including evenings and weekends.

‘Crisis assessment services’ are designed to provide people in crisis with rapid assessment and referral on to other acute mental health services where deemed appropriate (7). ‘Lifeguard Pharmacies’ offer people experiencing suicidal thoughts and/or domestic abuse discreet consultations with pharmacists, who can signpost them to appropriate community support (23).

‘Mental health crisis hubs’ focus on assessment, stabilisation and connecting individuals to appropriate support, while ‘Crisis cafés’, which are usually voluntary sector run and can be peer-led, provide informal spaces for support and connection (7,24). These walk-in services are intended to divert people from hospital emergency departments, which can be potentially distressing and unsuitable locations for assessment for people in crisis, associated with a higher risk of inpatient admission (25,26).

Discharge transition support

‘Discharge transition support’ models aim to assist individuals with transitioning from inpatient care to the community, promoting recovery, independence, and reducing readmissions. The ‘Transition to Recovery Program’ includes psychosocial education, practical assistance, symptom management, social support and connecting individuals with appropriate services (27,28). The ‘Peer Bridger Project’ involves peer workers who offer goal setting, skills teaching, emotional support and advocacy through one-to-one interactions and support groups for an average of 12 months around the time of discharge from inpatient care (29).

Outpatient-based crisis services

The ‘Whole-of-service stepped-care' model is a brief outpatient intervention approach for people with a “personality disorder” diagnosis. It offers immediate brief psychological intervention to people in crisis, followed by options for longer-term treatment (30).

Community services with crisis function

This category captures community-based mental health services which provide secondary mental health care generally and include some capacity for managing crises among people on their caseloads.

*Enhanced community mental health teams*

‘Enhanced community mental health teams’ (CMHT), also known as ‘extended hours’ teams, aim to proactively prevent crises and offer crisis intervention where needed, in addition to performing the standard ongoing case management functions typical of a standard CMHT (31).

*Early intervention models*

‘Early intervention models’ in mental health focus on specialist interventions, including detection, assessment, individualised treatment planning and psychosocial interventions, delivered by multidisciplinary teams. ‘Early intervention in psychosis’ (EIP) services support individuals aged 14-65 for up to three years after their first episode of psychosis. They offer assessment, pharmacological and psychological interventions, family support, employment/education assistance, help with social care issues, and crisis planning and intervention including rapid assessment, intensive home visits, and intensive case management (32,33). They aim to reduce treatment delays, promote recovery, avoid hospitalisation and prevent relapse (34–36).

*Enhanced case management models*

‘Enhanced case management’ models for adults include ‘Assertive community treatment’ (ACT), ‘Intensive case management’ (ICM), ‘Flexible assertive community treatment’ (FACT) and ‘Peer-delivered case management’. They focus on long-term care coordination to ensure individuals receive appropriate services, treatment adherence support and crisis management, including individualised crisis planning, emergency home visits, and on-call crisis support (37). These models involve multidisciplinary teams with small caseloads, providing intensive support in individuals’ home environments. In ACT, staff caseloads are shared whereas in ICM they are not. In FACT, care-coordinators manage individual caseloads but can also provide shared care at times of increased need (38,39). ACT and FACT teams can provide 24/7 support, whilst ICM teams typically operate during standard business hours (37). Peer-delivered case management involves peer workers alongside health care professionals (40–43).

### 1.2. Hospital-based alternatives

Models involving general hospital medical care

*Enhanced psychiatric liaison services*

‘Enhanced psychiatric liaison services’ aim to provide psychiatric assessment, treatment and support to people who are admitted to general hospitals or emergency departments with comorbid physical and mental health needs, including people in crisis. They ensure appropriate follow-up care post-discharge and are typically staffed by multidisciplinary teams. Enhanced models (including ‘Comprehensive’ and ‘Enhanced 24’ models e.g., ‘Rapid Assessment Interface Discharge’) offer more specialised care than standard psychiatric liaison services, increased psychiatric consultant input, and enhanced follow-up support (22,44–48), in part aiming to prevent avoidable inpatient admissions (47).

*Time-limited admission to general medical wards with specialist community eating disorder team input*

People with eating disorders may sometimes be admitted to general medical wards for medical stabilisation, with input from specialist community eating disorder teams. This approach is designed for people who have medical needs requiring inpatient care, including those who are too physically unwell for specialist eating disorder units (49,50).

Brief-stay crisis units

‘Brief-stay crisis units’ offer assessment, short-term treatment, and stabilisation for people in crisis outside of an inpatient setting, aiming to avoid inpatient mental health service admissions (51). They are generally linked to hospital emergency departments. The environment is less restrictive than inpatient psychiatric units, and length of stay typically ranges from a few hours to a few days (51). They include ‘Psychiatric observation units’, ‘Psychiatric decision units’, ‘Emergency psychiatric assessment, treating and healing (EmPATH) units’, ‘Psychiatric emergency service centres’, ‘23-hour crisis stabilisation units’ and ‘Behavioural assessment units’.

Inpatient psychiatric services

*Short-stay acute inpatient wards*

‘Short-stay acute inpatient psychiatric wards’ provide assessment, stabilisation and intensive interventions for people requiring intensive psychiatric care for a limited period of time, usually ranging from a day up to a week, with the aim of facilitating discharge home within the admission time period wherever possible (1,7). These contrast to standard inpatient mental health wards, which do not typically have a fixed length of stay (1).

*Inpatient psychiatric services with a specific therapeutic model*

‘Safewards’, ‘Star Wards’, ‘Talk 1^st^, the ‘Tidal Model’, the ‘Bradford Refocusing Model’, ‘Six Core Strategies’ and the ‘HOPES model’ are specific therapeutic models aimed at improving care in inpatient mental health settings (1,52–64). They prioritise person-centred care, collaboration, prevention of crises and minimisation of risk. These models emphasise therapeutic relationships, recovery-oriented environments and staff training. ‘Drug-free and minimal medication wards’ in Norway also offer an alternative therapeutic approach (65).

‘Brief Admission’ (BA) and ‘Preventative Admission’ (PA) are two distinct models which have been implemented on standard psychiatric wards, which aim to proactively reduce crises and inpatient admissions through contracting admissions. The BA self-referral model allows individuals with emotional instability and/or a history of repeated self-harm to hospitalise themselves in a pre-negotiated way, in line with a contract created with clinicians at a time of non-crisis (66). In contrast, the PA model involves pre-arranged admissions over a longer period of time (67). As such, these two service models are distinct from ‘short stay acute inpatient wards’ (described above).

*Inpatient wards for specific groups*

Specialist inpatient services are designed to meet the needs of specific groups such as people who are deaf (68) or people with specific psychiatric diagnoses (e.g., eating disorders, “personality disorders”, early psychosis). These services aim to provide specialised care to these groups, compared to the more generic care provided by standard inpatient wards. Inpatient services for people diagnosed with a “personality disorder” employ therapies like dialectical behavioural therapy or mentalisation-based treatment (69–71). Specialist eating disorder wards focus on medical stabilisation, nutritional rehabilitation, psychotherapy and developing effective coping skills (11,72). Early psychosis wards aim to provide timely intervention and comprehensive treatment (73,74).

### 1.3. Cross-setting approaches

These approaches can be applied across a range of settings and stages in care pathways and include elements that are expected to reduce dependence on acute care.

‘The Sanctuary model’, ‘Enabling Environments’, ‘Therapeutic Communities’ and ‘Democratic Therapeutic Communities’ (DTCs) are approaches that aim to create supportive environments for people experiencing mental health difficulties, including those in crisis. The ‘Sanctuary Model’ focuses on creating trauma-informed environments (63,75), whilst ‘Therapeutic Communities’ are structured environments based on shared values such as attachment and respect, promoting recovery through social relationships and activities (76). DTCs are a type of therapeutic community based on the principles of democratic decision making, communal sharing, permissiveness with consequences, and reality confrontation (77,78). ‘Enabling Environments’ similarly aim to prioritise positive social environments for recovery and community integration (79). Each of these approaches can be applied in a range of settings, including acute inpatient settings.

‘Need-adapted treatment’ focuses on providing personalised care tailored to individuals’ needs, guided by a psychotherapeutic and family-centred approach (80). It can be applied across different contexts, including those providing crisis support. Two examples of its implementation were identified - the ‘Swedish Parachute Project’ (81–83) and ‘Acute Psychosis Integrated Treatment’ (84,85).

‘Open Dialogue’, developed from need-adapted treatment, is a person-centred and network-centred approach to mental health care that involves systemic family therapy and psychodynamic principles. It emphasises open and transparent relationships between staff and service users as well as involving families and support networks, and there is a peer-supported variant (86,87). It has been implemented in a variety of contexts, including inpatient mental health settings (87).

‘Wraparound with intensive services’ similarly aims to engage multiple systems (e.g., health, social, education and youth justice services) to support and empower people aged 0-20 years old with complex needs and their families in the community, aiming to avoid out-of-home placements (88–90).

The ‘Trieste model’ in Italy is a whole-system approaches to organising care. It is a community-based approach to mental health care, including crisis support. It prioritises human rights, social inclusion and recovery. There are no locked doors and restrictive care is avoided (91,92).

Finally, there are a number of ‘Specialist consultancy’ models in operation that aim to promote alternative approaches to standard inpatient care and reduce inpatient admissions by offering consultation to relevant organisations. This consultancy may be provided by survivor-led, not-for-profit, statutory or private organisations (93–98).

## 2. Adult long-term inpatient care alternatives

Along with the array of services we identified which aim to offer support in times of acute crisis, service models were also identified that offer an alternative to standard long-term term hospital wards, including inpatient rehabilitation or forensic mental health care (see Supplementary Material 4). These services largely aim to reduce the length of hospitalisation, support people to reside in the community, or offer a therapeutic approach that differs substantially from standard long-term inpatient care. Though most secondary community mental health models have a role in avoidance of long-term hospitalisation, the focus here is on models that are more obviously intensive forms of support that could substitute for an inpatient admission. These service models were similarly categorised into community-based, hospital-based and cross-setting approaches. Further information, and associated outcome evidence identified, is provided for each model in Supplementary Material 2 and Supplementary Material 7.

### 2.1. Community-based alternatives

Intensive residential services

This category encompasses ‘Residential rehabilitation services’, and ‘Intensive supported housing’ models (99). Individuals typically move through the components of this pathway with the ultimate aim of successfully managing an independent tenancy and avoiding re-hospitalisation.

‘Residential rehabilitation services’ offer communal residential living for adults with chronic mental health conditions and significant impairment in social functioning whose needs cannot be well-served in the community (100,101). They represent a step-down from long-term inpatient mental health services. Residential rehabilitation services are staffed 24-hours a day and provide people with a high level of support, to support them to stay out of hospital (102). People typically stay for several years (99), however some shorter-term residential facilities also exist. Some residential rehabilitation services aim to be specialised for specific diagnostic groups, such as individuals with a primary diagnosis of an eating disorder or psychosis.

‘Intensive supported housing’ represents a step-down from 24-hour residential rehabilitation care, offering time-limited tenancies in shared or individual self-contained units with high-level support, sometimes including 24-hours a day staffing (100,101). Our typology only includes supported housing models which involve intensive support, with the aim of supporting people with severe and chronic mental health problems to maintain residence in the community and avoid hospitalisations (102). ‘Intensive supported housing’ can be offered with integrated care offered by the accommodation provider or with support outsourced and offered by external services (100,101). ‘Housing First’ and ‘Full Service Partnership’ represent two models of ‘supported housing with external intensive community support’ which typically serve communities experiencing or at-risk of homelessness and who also experience mental health difficulties (99,103–105).

Family placement schemes

Some family placement schemes can offer longer-term support to people with mental health difficulties who would otherwise not be able to manage living independently in the community. In the ‘Geel family foster care’ model, people of any age with various mental health difficulties are accommodated by foster families and are encouraged to participate in daily household activities and family life (106). The average length of stay with a Geel placement is approximately 30 years (106). ‘Healing Homes’, a similar model derived from Sweden, supports people with psychosis to live in host families for approximately 1-2 years (107). As well as offering support in an acute crisis (see Section *1.1.*) the ‘Shared Lives’ model also operates as an alternative for individuals requiring long-term placements (8).

Enhanced case management models

These models aim to avoid the need for long-term inpatient admissions by providing intensive support to people with complex mental health needs within the community. ‘Specialist community mental health rehabilitation teams’ are a variation of traditional CMHTs which offer specialised rehabilitation care in the community, often working with people in intensive residential services or ensuring safe transitions between settings (100,101). Designated care coordinators oversee service users’ progression through the rehabilitation pathway and liaise with other providers in the statutory and voluntary sectors (100,101). Likewise, ACT, FACT and intensive case management models, as described in *Section 1.1.,* can offer long-term enhanced case management support.

Discharge transition support

The ‘Peer Bridger Project’, described in *Section 1.1.*, often works with people who have been hospitalised for long periods of time or who have experienced repeated hospitalisations, aiming to support successful transitions from inpatient care back into the community (29).

### 2.2. Hospital-based alternatives

Inpatient psychiatric services

*Inpatient psychiatric services with a specific therapeutic model*

‘Hostel wards’ provide residential inpatient care for long-stay patients in inpatient mental health services who are unable to manage in the community and require 24/7 nursing care. Residents remain inpatients legally and staffing levels are similar to standard inpatient care settings. However, they are distinct from standard long-term wards in that the style of accommodation is more domestic, and residents are given a programme of domestic chores and self-care activities judged to be within their abilities (108).

As described in relation to acute settings in *Section 1.2.*, the ‘Safewards’ programme has also been implemented in medium- and long-term inpatient settings (109).

*Inpatient wards for people with specific psychiatric conditions*

Specialist longer-term inpatient wards may offer tailored care for specific clinical groups, such as people with “personality disorder”, or other groups with specific needs, such as deaf people. An example identified in our consultation was the Springbank Unit, an inpatient unit for women with a diagnosis of “borderline personality disorder”, which also aimed to implement less restrictive risk management procedures (e.g., by offering service users an optional conversation with staff in the place of a formal risk assessment checklist when leaving the ward) (70).

### 2.3. Cross-setting approaches

A range of cross-setting approaches were also identified in relation to long-term rehabilitation. Firstly, as described in *Section 1.3.*, ‘Therapeutic communities’ and ‘DTCs’ may also offer an alternative approach to standard longer term inpatient care, in both inpatient rehabilitation, residential and day hospital settings (6,76).

Second, as described in *Section 1.3.*, the ‘Trieste model’ also operates as a community alternative for longer term rehabilitation. For example, it provides people with supported housing, and there is a residential rehabilitation service which offers up to six months of support (110).

Third, as described above in S*ection 1.3.*, ‘Enabling Environments’ can be applied in a range of settings, including long-term rehabilitation settings for people with chronic mental health difficulties (79).

Finally, as described in *Section 1.3.*, there are ‘Specialist consultancy’ models which aim to provide expert consultancy to health care organisations and intensive intervention to individuals to avoid and reduce long-term mental health hospitalisations (93,95–98).

## 3. Adult long-term inpatient care alternatives: forensic

A range of alternative service models were identified which operate in forensic settings across a variety of countries (see Supplementary Material 4). Some of the service models identified below represent components of the Offender Personality Disorder (OPD) pathway in the UK, which operates exclusively in forensic contexts. This aims to offer a pathway of psychologically informed services for offenders with likely “severe personality disorder” (111). Further information, and identified outcome evidence, for each model is provided in Supplementary Material 2 and Supplementary Material 7.

### 3.1. Community-based alternatives

Residential community care

Similar to the long-term rehabilitation findings above (see *Section 2.1.*), residential community care also serves offending populations in the form of either ‘intensive residential services’ or ‘secure accommodation’.

‘Intensive residential service’ options identified overlap with those for non-forensic populations. They include ‘Residential rehabilitation services’, ‘Integrated supported housing’ and ‘Housing with external intensive community support’ models (e.g., ‘Housing First’ and ‘Full Service Partnership’).

‘Housing and Accommodation Support Services’ (HASS) are specific to forensic populations. They are an ‘integrated supported housing’ component of the OPD pathway, specifically offering support and accommodation to people meeting OPD pathway criteria on probation who have been released from prison and other secure health care settings (e.g., inpatient care), or those who are transitioning on from other residential settings housing ex-offenders in the community (112,113).

‘Residences for the Execution of Security Measures’ (REMS), based in Italy, are a type of secure accommodation offering an alternative residential approach for forensic populations who have been given a custodial order. REMS are intended to be a therapeutic space with an aim for rehabilitation, without the involvement of police officers (114).

As described above in *Section 1.1.* and *Section 2.1.*, ‘Shared Lives’ is a family placement scheme which can offer support to offenders as well as non-offending populations (115).

Enhanced case management models

‘Forensic ACT’ teams are an adaptation of the general ACT model, involving co-ordination with criminal justice entities, provisions of legal advocacy and assistance with applying for financial support, and with a primary aim of reducing criminal justice involvement and reoffending (116). Forensic ACT teams operate in a similar manner to ACT teams, but with the added provision of forensic psychiatrists and probation officers usually (117).

Intensive Intervention and Risk Management Services (IIRMS)

‘IIRMS’ are a component of the OPD pathway which may be based in either prisons or the community. They offer psychologically-informed case management, working with individuals who are being released from a prison or secure environment into the community. IIRMS provide both in-reach to prison settings and outreach to community settings and aim to enhance skills and self-management necessary for a successful resettlement (118).

‘Transitional support and liaison services’ are a specialist type of IIRMS which aim to help people access support offered by statutory and voluntary organisations, both during and after their transition from custody to the community (119). The support duration is typically shorter, typically spanning just a few months, compared to standard IIRMS support, which typically extends for a year or longer (119).

### 3.2. Hospital-based alternatives

Inpatient services

As described in relation to acute inpatient settings (*Section 1.2.),* some forensic inpatient settings have employed therapeutic models such as the ‘Tidal Model’ (61), ‘Six Core Strategies’ (120,121) and ‘Safewards’ (57,109,122). Secure wards also exist which specifically accept offenders from certain groups, for example, deaf people or people with a diagnosis of “personality disorder”.

Another forensic inpatient model distinct to standard care are ‘Ter Beschikking Stelling’ (TBS) hospitals in the Netherlands. Dutch courts can impose combination verdicts on offenders with severe mental health difficulties and high risk of recidivism (123). These offenders can be detained in specialist TBS hospitals, which aim to reintegrate them back into the community (123). In contrast to forensic hospitals in the UK, TBS hospitals have wards with different levels of security within the same institution, offering greater continuity of care when people move between them, and are less restrictive (e.g., offering more people unsupervised leave and family visits) (124).

### 3.3. Cross-setting approaches

Finally, there are also a range of approaches which can be applied across multiple settings. As described above in relation to acute contexts (*Section 1.3.)* and long-term rehabilitation (*Section 2.3.),* ‘Therapeutic Communities’ have been implemented in forensic settings, such as forensic inpatient and prison services, as have ‘DTCs’, which offer a whole-system approach to rehabilitation (77).

A further approach, ‘Psychologically Informed Planned Environments’ (PIPES) are another component of the OPD pathway. Whilst PIPES are primarily adopted in prison settings, they are also evidenced in alternative settings, such as community-based hostels for individuals who have recently been released from prison. PIPES offer training to staff to facilitate a safe and supportive environment according to several key principles: the development of an ‘enabling environment’; structured groups between staff and residents; socially creative sessions and training; and the provision of supervision and reflective spaces for staff (125). In a similar vein, as described above in relation to acute care (*Section 1.3.*) and long-term rehabilitation *(Section 2.3.*), ‘Enabling Environments’ can be found in a range of settings, including forensic services.

Some OPD services can operate in multiple settings. ‘OPD treatment services’ may be offered either in secure settings or in the community for individuals who are subject to probation supervision. For example, these may include Mentalisation Based Treatment services or Male-Trauma Recovery Empowerment Model services (126). ‘Psychologically enhanced resettlement services’, another component of the OPD pathway, also offer support either within prisons or within the community, and aim to support the transition-in, graduation, and transition-out of open conditions (127).

Finally, as listed in relation to the acute crisis care (*Section 1.3.*), the ‘Sanctuary Model’ also operates in forensic settings (63), as do some ‘Specialist consultancies’ and the ‘Trieste model’.

## 4. CYP alternatives: Acute, long-term and forensic

A range of service models were also identified for CYP (see Supplementary Material 5). Some were unique to this population, whilst others were also identified during the adult typology mapping and, as such, are described in greater detail in prior sections. Further information, and identified outcome evidence, is provided for each model in Supplementary Material 2 and Supplementary Material 7.

### 4.1. Community-based alternatives

Residential services

Similar to provision for adults – although not as widely implemented – crisis houses were identified for CYP aged 11 and over. Soteria houses and ‘Shared Lives’ are adult crisis residential models that can also accept CYP aged 16 and over (8).

A family placement scheme specific to CYP (aged three and over) is ‘Therapeutic foster care’, which involves structured therapy within a foster family setting, usually for 6-9 months (128–130). It can support forensic and non-forensic CYP populations and can be initiated at a time of crisis (130). It aims to provide CYP in foster care and youth justice programs with an alternative to more restrictive placements (130). The ‘Geel family foster care’ model is another family placement scheme, previously described for adults in *Section 2.1.,* that can also provide long-term support to CYP of all ages.

Two further residential models of secure accommodation for CYP populations include ‘Secure children’s homes’ and ‘Secure training centres’ (STCs). Secure children’s homes are specifically tailored for CYP aged 10 to 17 years and offer full residential care, formal on-site education, and health care provision. On-site mental health professionals ultimately aim to successfully re-integrate service users back into the community (131,132). They accept CYP who are sentenced or on remand through the justice system, or placed due to local authority concerns that no other type of placement could keep them safe (131). Whereas STCs are detention facilities for CYP aged 12-17 years old who have been convicted of a criminal offence or are awaiting trial only. They are usually operated by private companies and their ethos is more punitive than in secure children’s homes, with lower staff ratios and less extensively trained staff (133).

As detailed in the adult section above, ‘Residential rehabilitation services’ and ‘Full Service Partnership’ - a ‘Housing with external intensive community support’ model - can also support CYP.

Acute day units

Both ‘General acute ADUs’ and ‘Specialist ADUs’ for eating disorders also exist for CYP, where support is tailored to the needs of CYP at the time of a crisis. ADU programmes for CYP may provide a combination of individual therapy, family therapy, medication management and educational services (129,134,135).

Home-based crisis services

‘CRHTTs’ and ‘Other intensive home treatment models’ can also provide support to CYP, sometimes with dedicated teams for CYP. A model specifically for CYP aged 11-18 years old is ‘Intensive home treatment with optional brief inpatient admission’. This involves providing a CRHTT approach with an optional short admission to a psychiatric intensive care unit, where the family is supported by the same professionals as in the community (136). Other CYP-specific models also include ‘Homebuilders’ and ‘Enhanced Homebuilders’, which offer short-term, intensive, home-based services aimed at resolving crises, improving family relationships and connecting families with necessary support (including financial support and respite care) (137). ‘Enhanced Homebuilders’ focuses on cultural competence and addressing violence (137).

Emergency service linked models

‘Police and/or ambulance street triage teams’ and ‘Clinician-only mobile crisis units’, previously described, can also provide support to CYP (18).

Drop-in crisis services

Similar to the ‘Crisis café’ model identified for adult populations (see *Section 1.1.*), a few examples of crisis cafés for CYP were identified (157), alongside ‘Crisis assessment services’ and ‘Mental health crisis hubs’ (138,139).

Discharge transition support

‘Supported Discharge Service’ and ‘Hot-BITS’ models are discharge transition support models for CYP. Both models involve the provision of psychiatric reviews, psychological interventions, support with education, social issues and physical health, and out-of-hours support (140–143) to facilitate successful transition from inpatient care to the community.

Outpatient-based crisis services

The ‘Behavioural health crisis care clinic’ is a brief outpatient intervention model for CYP providing assessment, safety planning, coping skills and coordinating further care for those experiencing suicidal thoughts or recent suicide attempts (144). The ‘Whole-of-service stepped-care' model, previously described for adults with a diagnosis of “borderline personality disorder”, can also support CYP aged 12 and over (30).

Community services with crisis function

*Enhanced case management models*

‘Crisis case management’ (CCM) is an enhanced case management model for CYP aged 10 and over. It provides shorter-term crisis support for families and additional resources like respite care (145). ACT has been used with CYP aged 10 and over, and FACT with CYP aged 12 and over (38,146,147).

*Early intervention models*

EIP services can accept CYP aged 14 years old and over.

*Adolescent forensic community services*

‘Adolescent forensic community services’ provide assessment and intervention for young people with complex mental health needs and a high risk of offending behaviour, and sometimes also provide in-reach to juvenile secure estates and children’s homes (129). They aim in part to avoid inpatient admissions and shorten inpatient lengths of stay (129).

### 4.2. Hospital-based alternatives

Models involving general hospital medical care

*Enhanced psychiatric liaison services*

The ‘Enhanced psychiatric liaison models’ (‘Enhanced 24’ and ‘Comprehensive’ models) described above serve both adults and CYP aged 16 and over (148). ‘Paediatric psychiatric liaison’ services specifically provide support to CYP of all ages (149).

*Time-limited admission to general medical wards with specialist community eating disorder team input*

As described above in the adult section, this model also exists for CYP with eating disorders requiring medical stabilisation (150).

Brief-stay crisis units

Some types of ‘Brief-stay crisis units’ can also provide care to CYP, including ‘Psychiatric emergency service centres’, ‘23-hour crisis stabilisation units’ and ‘Behavioural assessment units’ (151,152).

Short-stay acute inpatient wards

‘Short-stay acute inpatient psychiatric wards’ also exist which can accept CYP aged 16 and over (1).

Inpatient psychiatric services with a specific therapeutic model

Some therapeutic models implemented in adult inpatient services have also been implemented in inpatient services for CYP, including ‘Safewards’ (153,154), ‘Six Core Strategies’ and the ‘HOPES’ model (64). Furthermore, an evaluation of the ‘BA’ model described in *Section 1.2.,* adapted for adolescents, is currently in progress (155).

Inpatient wards for specific groups

As for adults, there are ‘Specialist eating disorder wards’ for CYP, and ‘Early psychosis wards’ which can accept CYP aged 16 and over. Specialist wards also exist for deaf CYP (156). There are also inpatient wards specifically for children under the age of 13, which may provide schooling, support to parents/carers and age-appropriate therapeutic activities (157).

### 4.3. Cross-setting approaches

Multisystemic therapy is a cross-setting model specific to CYP aged 10 and over. It aims to engage multiple systems in order to provide intensive support to CYP and their families in their natural environments (129). It has been implemented with both forensic and non-forensic populations. ‘Wraparound with intensive services’, previously described, is for CYP aged 0-20 (90). Likewise, ‘Open Dialogue’ (but not peer-supported open dialogue), ‘Therapeutic Communities’, ‘DTCs’, the ‘Sanctuary model’, ‘Enabling Environments’, ‘Need-adapted Treatment’, and the ‘Trieste model’ have also been implemented for CYP populations.

## References

1. Lloyd-Evans B, Slade M, Jagielska D, Johnson S. Residential alternatives to acute psychiatric hospital admission: systematic review. Br J Psychiatry. 2009 Aug;195(2):109–17.

2. Cooke A, McNicholas S, Rose A. Women and Power: Drayton Park Women’s Crisis House. In: Inside Out, Outside In: Transforming Mental Health Practices [Internet]. PCCS Books; 2019. Available from: https://www.candi.nhs.uk/sites/default/files/Women%20and%20Power%20-%20The%20Drayton%20Park%20Women%27s%20Crisis%20House.pdf

3. Killaspy H, Dalton J, McNicholas S, Johnson S. Drayton Park, an alternative to hospital admission for women in acute mental health crisis. Psychiatr bull. 2000 Mar;24(3):101–4.

4. Howard L, Flach C, Leese M, Byford S, Killaspy H, Cole L, et al. Effectiveness and cost-effectiveness of admissions to women’s crisis houses compared with traditional psychiatric wards: pilot patient-preference randomised controlled trial. Br J Psychiatry. 2010 Aug;197(S53):s32–40.

5. Johnson S, Bingham C, Billings J, Pilling S, Morant N, Bebbington P, et al. Women’s experiences of admission to a crisis house and to acute hospital wards: A qualitative study. Journal of Mental Health. 2004 Jun;13(3):247–62.

6. Stupak R, Dobroczyński B. From Mental Health Industry to Humane Care. Suggestions for an Alternative Systemic Approach to Distress. IJERPH. 2021 Jun 20;18(12):6625.

7. Dalton-Locke C, Johnson S, Harju-Seppänen J, Lyons N, Sheridan Rains L, Stuart R, et al. Emerging models and trends in mental health crisis care in England: a national investigation of crisis care systems. BMC Health Serv Res. 2021 Dec;21(1):1174.

8. Harflett N, Jennings Y. Evaluation of the Shared Lives Mental Health Project [Internet]. Bath, United Kingdom: Shared Lives Plus; 2016. Available from: https://www.ndti.org.uk/assets/files/Final_NDTi_Cabinet_Office_SLP_MH_Eval_Report.pdf

9. Marshall M, Sledge W, Wiersma D, Crowther R, Kluiter H, Bond GR, et al. Systematic reviews of the effectiveness of day care for people with severe mental disorders: (1) Acute day hospital versus admission; (2) Vocational rehabilitation; (3) Day hospital versus outpatient care. Health Technol Assess [Internet]. 2001 [cited 2023 Jun 23];5(21). Available from: https://www.journalslibrary.nihr.ac.uk/hta/hta5210/

10. Morant N, Davidson M, Wackett J, Lamb D, Pinfold V, Smith D, et al. Acute day units for mental health crises: a qualitative study of service user and staff views and experiences. BMC Psychiatry. 2021 Dec;21(1):146.

11. Madden S, Hay P, Touyz S. Systematic review of evidence for different treatment settings in anorexia nervosa. WJP. 2015;5(1):147.

12. Bateman A, Fonagy P. Effectiveness of Partial Hospitalization in the Treatment of Borderline Personality Disorder: A Randomized Controlled Trial. AJP. 1999 Oct;156(10):1563–9.

13. Antonsen BT, Klungsøyr O, Kamps A, Hummelen B, Johansen MS, Pedersen G, et al. Step-down versus outpatient psychotherapeutic treatment for personality disorders: 6-year follow-up of the Ullevål personality project. BMC Psychiatry. 2014 Dec;14(1):119.

14. Johnson S. Crisis resolution and home treatment teams: an evolving model. Adv psychiatr treat. 2013 Mar;19(2):115–23.

15. Lloyd-Evans B, Bond GR, Ruud T, Ivanecka A, Gray R, Osborn D, et al. Development of a measure of model fidelity for mental health Crisis Resolution Teams. BMC Psychiatry. 2016 Dec;16(1):427.

16. Boege I, Schepker R, Fegert JM. Vom Hometreatment zur stationsäquivalenten Behandlung (StäB): Ein systematischer Review aufsuchender Behandlung in Deutschland. Zeitschrift für Kinder- und Jugendpsychiatrie und Psychotherapie. 2020 Sep 1;48(5):393–406.

17. Lay B, Blanz B, Schmidt MH. Effectiveness of home treatment in children and adolescents with externalizing psychiatric disorders. European Child & Adolescent Psychiatry. 2001 Mar;10(S1):S80–90.

18. Marcus N, Stergiopoulos V. Re‐examining mental health crisis intervention: A rapid review comparing outcomes across police, co‐responder and non‐police models. Health Social Care Comm. 2022 Sep;30(5):1665–79.

19. Preyde M, Frensch K, Cameron G, Hazineh L, Riosa PB. Mental Health Outcomes of Children and Youth Accessing Residential Programs or a Home-Based Alternative. Social Work in Mental Health. 2010 Dec 30;9(1):1–21.

20. Preyde M, Frensch K, Cameron G, White S, Penny R, Lazure K. Long-term Outcomes of Children and Youth accessing Residential or Intensive Home-based Treatment: Three year follow up. J Child Fam Stud. 2011 Oct;20(5):660–8.

21. Schmidt MH, Lay B, Göpel C, Naab S, Blanz B. Home treatment for children and adolescents with psychiatric disorders. Eur Child Adolesc Psychiatry. 2006 Aug;15(5):265–76.

22. Odejimi O, Bagchi D, Tadros G. Typology of psychiatric emergency services in the United Kingdom: a narrative literature review. BMC Psychiatry. 2020 Dec;20(1):587.

23. Lifeguard Pharmacy. Lifeguard Pharmacy: Supportive signposting for people in danger from themselves or others [Internet]. 2023. Available from: https://lifeguardpharmacy.lincoln.ac.uk/

24. Health Innovation Network, NHS England. Evaluating NHS Mental Health Crisis Hubs in London: Final Report [Internet]. 2022. Available from: https://healthinnovationnetwork.com/wp-content/uploads/2022/11/MH-Crisis-Hubs-Evaluation-Final-Report.pdf

25. Johnson S, Dalton‐Locke C, Baker J, Hanlon C, Salisbury TT, Fossey M, et al. Acute psychiatric care: approaches to increasing the range of services and improving access and quality of care. World Psychiatry. 2022 Jun;21(2):220–36.

26. Cotton MA, Johnson S, Bindman J, Sandor A, White IR, Thornicroft G, et al. An investigation of factors associated with psychiatric hospital admission despite the presence of crisis resolution teams. BMC Psychiatry. 2007 Dec;7(1):52.

27. Australian National University. Evaluation of Transition to Recovery (TRec) Program. [Internet]. 2015. Available from: https://www.wcs.org.au/services/mental-health/transition-to-recovery/anu-evaluation-of-the-transition-to-recovery-program/

28. Woden Community Service. Woden Community Service [Internet]. Available from: https://www.wcs.org.au

29. New York Association of Psychiatric Rehabilitation Services Inc. Peer Bridger Project [Internet]. Available from: https://www.nyaprs.org/peer-bridger

30. Grenyer BFS, Lewis KL, Fanaian M, Kotze B. Treatment of personality disorder using a whole of service stepped care approach: A cluster randomized controlled trial. van Wouwe JP, editor. PLoS ONE. 2018 Nov 6;13(11):e0206472.

31. Rosen A, Clenaghan P, Emerton F, Richards S. Integration of the crisis resolution function within community mental health teams. In: Johnson S, Needle J, Bindman JP, Thornicroft G, editors. Crisis Resolution and Home Treatment in Mental Health [Internet]. 1st ed. Cambridge University Press; 2008 [cited 2023 Aug 8]. p. 235–50. Available from: https://www.cambridge.org/core/product/identifier/CBO9780511543906A033/type/book_part

32. Puntis S, Minichino A, De Crescenzo F, Harrison R, Cipriani A, Lennox B. Specialised early intervention teams for recent-onset psychosis. Cochrane Schizophrenia Group, editor. Cochrane Database of Systematic Reviews [Internet]. 2020 Nov 2 [cited 2024 Jun 13];2021(2). Available from: http://doi.wiley.com/10.1002/14651858.CD013288.pub2

33. O’Connell N, O’Connor K, McGrath D, Vagge L, Mockler D, Jennings R, et al. Early Intervention in Psychosis services: A systematic review and narrative synthesis of the barriers and facilitators to implementation. Eur Psychiatr. 2022;65(1):e2.

34. Skalli L, Nicole L. Programmes pour premiers épisodes psychotiques : une revue systématique de la littérature. L’Encéphale. 2011 May;37:S66–76.

35. Bertelsen M, Jeppesen P, Petersen L, Thorup A, Øhlenschlæger J, le Quach P, et al. Five-Year Follow-up of a Randomized Multicenter Trial of Intensive Early Intervention vs Standard Treatment for Patients With a First Episode of Psychotic Illness: The OPUS Trial. Arch Gen Psychiatry. 2008 Jul 7;65(7):762.

36. McCrone P, Craig TKJ, Power P, Garety PA. Cost-effectiveness of an early intervention service for people with psychosis. Br J Psychiatry. 2010 May;196(5):377–82.

37. Rubin A, Springer DW, Trawver K, editors. Psychosocial treatment of schizophrenia: clinician’s guide to evidence-based practice. Hoboken, N.J: John Wiley & Sons; 2010. 388 p. (Clinician’s guide to evidence-based practice series).

38. Conrad N. Hilton Foundation, The National Center on Family Homelessness, National Alliance to End Homelessness, ZERO TO THREE: National Center for Infants, Toddlers and Families. Step by Step: A Comprehensive Approach to Case Management [Internet]. 2011. Available from: https://www.air.org/sites/default/files/March%202011%20-%20Step%20by%20Step%20-%20A%20Comprehensive%20Approach%20to%20Case%20Management.pdf

39. Sood L, Owen A, Onyon R, Sharma A, Nigriello J, Markham D, et al. Flexible assertive community treatment (FACT) model in specialist psychosis teams: An evaluation. BJPsych Bull. 2017 Aug;41(4):192–6.

40. Wright-Berryman JL, McGuire AB, Salyers MP. A Review of Consumer-Provided Services on Assertive Community Treatment and Intensive Case Management Teams: Implications for Future Research and Practice. J Am Psychiatr Nurses Assoc. 2011 Jan;17(1):37–44.

41. Solomon P, Draine J. One-year outcomes of a randomized trial of consumer case management. Evaluation and Program Planning. 1995 Apr;18(2):117–27.

42. Sells D, Davidson L, Jewell C, Falzer P, Rowe M. The Treatment Relationship in Peer-Based and Regular Case Management for Clients With Severe Mental Illness. PS. 2006 Aug;57(8):1179–84.

43. Clarke GN, Herinckx HA, Kinney RF, Paulson RI, Cutler DL, Lewis K, et al. Psychiatric hospitalizations, arrests, emergency room visits, and homelessness of clients with serious and persistent mental illness: findings from a randomized trial of two ACT programs vs. usual care. Mental Health Services Research. 2000;2(3):155–64.

44. Evans R, Connell J, Ablard S, Rimmer M, O’Keeffe C, Mason S. The impact of different liaison psychiatry models on the emergency department: A systematic review of the international evidence. Journal of Psychosomatic Research. 2019 Apr;119:53–64.

45. Walker A, Barrett JR, Lee W, West RM, Guthrie E, Trigwell P, et al. Organisation and delivery of liaison psychiatry services in general hospitals in England: results of a national survey. BMJ Open. 2018 Aug;8(8):e023091.

46. Jasmin K, Walker A, Guthrie E, Trigwell P, Quirk A, Hewison J, et al. Integrated liaison psychiatry services in England: a qualitative study of the views of liaison practitioners and acute hospital staffs from four distinctly different kinds of liaison service. BMC Health Serv Res. 2019 Dec;19(1):522.

47. Becker L, Saunders R, Hardy R, Pilling S. The RAID model of liaison psychiatry. Report on the evaluation of four pilot services in east London [Internet]. UCL Partners; 2016. Available from: https://www.ucl.ac.uk/pals/sites/pals/files/uclp_raid_evaluation_report.pdf

48. Royal College of Psychiatrists. Liaison psychiatry services [Internet]. 2018. Available from: https://www.rcpsych.ac.uk/mental-health/treatments-and-wellbeing/liaison-psychiatry-services

49. NHS England, NICE, National Collaborating Centre for Mental Health. Adult eating disorders: community, inpatient and intensive day patient care. Guidance for commissioners and providers. [Internet]. 2019. Available from: https://www.england.nhs.uk/wp-content/uploads/2019/08/aed-guidance.pdf

50. Royal College of Psychiatrists. Medical Emergencies in Eating Disorders: Guidance on Recognition and Management. [Internet]. 2023. Available from: https://www.rcpsych.ac.uk/docs/default-source/improving-care/better-mh-policy/college-reports/college-report-cr233-medical-emergencies-in-eating-disorders-(meed)-guidance.pdf?sfvrsn=2d327483_55

51. Anderson K, Goldsmith LP, Lomani J, Ali Z, Clarke G, Crowe C, et al. Short-stay crisis units for mental health patients on crisis care pathways: systematic review and meta-analysis. BJPsych open. 2022 Jul;8(4):e144.

52. Bowers L. Safewards: a new model of conflict and containment on psychiatric wards: Safewards: description of the model. J Psychiatr Ment Health Nurs. 2014 Aug;21(6):499–508.

53. Huckshorn K. Six Core Strategies for Reducing Seclusion and Restraint Use [Internet]. National Association of State Mental Health Program Directors (NASMHPD); 2006. Available from: https://www.nasmhpd.org/sites/default/files/2022-08/Consolidated%2520Six%2520Core%2520Strategies%2520Document.pdf

54. Simpson A, Janner M. Star Wards Survey Report 2009/10 [Internet]. City Research Online; 2019. Available from: https://openaccess.city.ac.uk/id/eprint/7246/1/Star%20Wards%20Survey%20Report%20_Final%2005052010_V2.pdf

55. Finch K, Lawrence D, Williams MO, Thompson AR, Hartwright C. A Systematic Review of the Effectiveness of Safewards: Has Enthusiasm Exceeded Evidence? Issues in Mental Health Nursing. 2022 Feb 1;43(2):119–36.

56. Ward‐Stockham K, Kapp S, Jarden R, Gerdtz M, Daniel C. Effect of Safewards on reducing conflict and containment and the experiences of staff and consumers: A mixed‐methods systematic review. Int J Mental Health Nurs. 2022 Feb;31(1):199–221.

57. Mullen A, Browne G, Hamilton B, Skinner S, Happell B. Safewards: An integrative review of the literature within inpatient and forensic mental health units. Int J Mental Health Nurs. 2022 Oct;31(5):1090–108.

58. Gordon W, Morton T, Brooks G. Launching the Tidal Model: evaluating the evidence: Launching the Tidal Model. Journal of Psychiatric and Mental Health Nursing. 2005 Dec;12(6):703–12.

59. Berger JL. Incorporation of the tidal model into the interdisciplinary plan of care - a program quality improvement project. J Psychiatr Ment Health Nurs. 2006 Aug;13(4):464–7.

60. Stevenson C, Fletcher E. The Tidal Model: the questions answered. Mental Health Practice. 2002 May;5(8):29–37.

61. Cook NR, Phillips BN, Sadler D. The tidal model as experienced by patients and nurses in a regional forensic unit. J Psychiatr Ment Health Nurs. 2005 Oct;12(5):536–40.

62. Bowles N, Dodds P. The use of refocusing in acute psychiatric care. Nursing Times. 2002;98(22):44–5.

63. Saunders KRK, McGuinness E, Barnett P, Foye U, Sears J, Carlisle S, et al. A scoping review of trauma informed approaches in acute, crisis, emergency, and residential mental health care. BMC Psychiatry. 2023 Aug 7;23(1):567.

64. Mersey Care NHS Foundation Trust website [Internet]. n.d. Available from: https://www.merseycare.nhs.uk/hopes-model

65. Nyttingnes O, Rugkåsa J. The Introduction of Medication-Free Mental Health Services in Norway: An Analysis of the Framing and Impact of Arguments From Different Standpoints. Front Psychiatry. 2021 Jul 19;12:685024.

66. Westling S, Daukantaite D, Liljedahl SI, Oh Y, Westrin Å, Flyckt L, et al. Effect of Brief Admission to Hospital by Self-referral for Individuals Who Self-harm and Are at Risk of Suicide: A Randomized Clinical Trial. JAMA Netw Open. 2019 Jun 7;2(6):e195463.

67. Koekkoek B, van der Snoek R, Oosterwijk K, van Meijel B. Preventive Psychiatric Admission for Patients With Borderline Personality Disorder: A Pilot Study. Perspectives in Psychiatric Care. 2010 Apr;46(2):127–34.

68. Souza R, Palmer L, Tarant E, Kaselionyte J, editors. Standards for Adult Inpatient Mental Health Services for Deaf People [Internet]. The Royal College of Psychiatrists; 2015. Available from: https://www.rcpsych.ac.uk/docs/default-source/improving-care/ccqi/quality-networks/deaf-services-qnmhd/qnmhd_standards_2nd_edition_2015.pdf?sfvrsn=b9b9be33_2

69. Cygnet Health Website [Internet]. n.d. Available from: https://www.cygnethealth.co.uk/services/personality-disorder/

70. Yue AC, Philbey AW, Crawford OA, Zimbron J. The Impact of Stopping Risk Assessment Checklists at a Specialist Personality Disorder Unit. Cureus [Internet]. 2023 Jan 18 [cited 2023 Jun 23]; Available from: https://www.cureus.com/articles/106062-the-impact-of-stopping-risk-assessment-checklists-at-a-specialist-personality-disorder-unit

71. West London NHS Trust Website [Internet]. n.d. Available from: https://www.westlondon.nhs.uk/our-services/adult/mental-health-services/cassel-hospital

72. Kotilahti E, West M, Isomaa R, Karhunen L, Rocks T, Ruusunen A. Treatment interventions for Severe and Enduring Eating Disorders: Systematic review. Int J Eat Disord. 2020 Aug;53(8):1280–302.

73. Siebert S, Leopold K, Baumgardt J, von Hardenberg LS, Burkhardt E, Bechdolf A. Specialized inpatient treatment for young people with early psychosis: acute-treatment and 12-month results. Eur Arch Psychiatry Clin Neurosci. 2022 Oct;272(7):1–14.

74. CAMH. Crisis and Critical Care Unit 5 Early Psychosis Unit (EPU - CCC5) [Internet]. n.d. Available from: https://www.camh.ca/en/your-care/programs-and-services/crisis-and-critical-care-unit-5#:~:text=The%20Early%20Psychosis%20Unit%20(EPU)%20is%20a%20specialist%20inpatient%20unit,mental%20health%20issues%20with%20psychosis.

75. The Sanctuary Institute [Internet]. Available from: https://www.thesanctuaryinstitute.org/about-us/the-sanctuary-model/

76. The Consortium for Therapeutic Communities. The Consortium for Therapeutic Communities [Internet]. 2022. Available from: https://therapeuticcommunities.org/

77. Warren F. Therapeutic communities. In: Brown JM, Campbell EA, editors. The Cambridge Handbook of Forensic Psychology [Internet]. 1st ed. Cambridge University Press; 2010 [cited 2023 Jun 23]. p. 423–33. Available from: https://www.cambridge.org/core/product/identifier/9780511730290%23c87809-6482/type/book_part

78. Norton K, Bloom SL. The Art and Challenges of Long-Term and Short-Term Democratic Therapeutic Communities. Psychiatr Q. 2004;75(3):249–61.

79. Royal College of Psychiatrists. Enabling Environments (EE) [Internet]. 2023. Available from: https://www.rcpsych.ac.uk/improving-care/ccqi/quality-networks-accreditation/enabling-environments-network-ee

80. Alanen YO, Lehtinen K, Räkköläinen V, Aaltonen J. Need-adapted treatment of new schizophrenic patients: experiences and results of the Turku Project. Acta Psychiatr Scand. 1991 May;83(5):363–72.

81. Cullberg J. Integrating intensive psychosocial therapy and low dose medical treatment in a total material of first episode psychotic patients compared to “treatment as usual” a 3 year follow-up. Med Arh. 1999;53(3):167–70.

82. Cullberg J, Levander S, Holmqvist R, Mattsson M, Wieselgren IM. One-year outcome in first episode psychosis patients in the Swedish Parachute project: One-year outcome in first episode psychosis patients. Acta Psychiatrica Scandinavica. 2002 Oct;106(4):276–85.

83. Cullberg J, Mattsson M, Levander S, Holmqvist R, Tomsmark L, Elingfors C, et al. Treatment costs and clinical outcome for first episode schizophrenia patients: a 3-year follow-up of the Swedish “Parachute Project” and Two Comparison Groups. Acta Psychiatr Scand. 2006 Oct;114(4):274–81.

84. Lehtinen K. Need-adapted treatment of schizophrenia: a five-year follow-up study from the Turku project. Acta Psychiatr Scand. 1993 Feb;87(2):96–101.

85. Lehtinen V, Aaltonen J, Koffert T, Räkköläinen V, Syvälahti E. Two-year outcome in first-episode psychosis treated according to an integrated model. Is immediate neuroleptisation always needed? Eur psychiatr. 2000 Aug;15(5):312–20.

86. Lorenz-Artz K, Bierbooms J, Bongers I. Introducing Peer-supported Open Dialogue in changing mental health care. Front Psychol. 2023 Jan 18;13:1056071.

87. Pilling S, Clarke K, Parker G, James K, Landau S, Weaver T, et al. Open Dialogue compared to treatment as usual for adults experiencing a mental health crisis: Protocol for the ODDESSI multi-site cluster randomised controlled trial. Contemporary Clinical Trials. 2022 Feb;113:106664.

88. Henggeler SW, Rowland MD, Randall J, Ward DM, Pickrel SG, Cunningham PB, et al. Home-Based Multisystemic Therapy as an Alternative to the Hospitalization of Youths in Psychiatric Crisis: Clinical Outcomes. Journal of the American Academy of Child & Adolescent Psychiatry. 1999 Nov;38(11):1331–9.

89. Henggeler SW, Rowland MD, Halliday‐Boykins C, Sheidow AJ, Ward DM, Randall J, et al. One‐Year Follow‐up of Multisystemic Therapy as an Alternative to the Hospitalization of Youths in Psychiatric Crisis. Journal of the American Academy of Child & Adolescent Psychiatry. 2003 May;42(5):543–51.

90. Bruns EJ, Pullmann MD, Sather A, Brinson RD, Ramey M. Effectiveness of Wraparound Versus Case Management for Children and Adolescents: Results of a Randomized Study. Adm Policy Ment Health. 2015 May;42(3):309–22.

91. Sashidharan SP. Why Trieste matters. Br J Psychiatry. 2022 Feb;220(2):52–3.

92. Living Well UK. Community Mental Healthcare: TRIESTE, ITALY [Internet]. 2022. Available from: https://www.livingwellsystems.uk/trieste

93. Beam Consultancy. Beam Consultancy: Clinical Expertise and Lived Experience in the areas of potentially lethal self harm, suicidality and complex interpersonal dynamics, often described as ‘personality disorder’ [Internet]. 2023. Available from: https://www.beamconsultancy.co.uk

94. Leeds Survivor Led Crisis Services: Trustees’ Report and Financial Statements for the Year Ended 31 March 2018 [Internet]. Leeds Survivor Led Crisis Services; 2018. Available from: https://register-of-charities.charitycommission.gov.uk/charity-search?p_p_id=uk_gov_ccew_onereg_charitydetails_web_portlet_CharityDetailsPortlet&p_p_lifecycle=2&p_p_state=maximized&p_p_mode=view&p_p_resource_id=%2Faccounts-resource&p_p_cacheability=cacheLevelPage&_uk_gov_ccew_onereg_charitydetails_web_portlet_CharityDetailsPortlet_objectiveId=A9335399&_uk_gov_ccew_onereg_charitydetails_web_portlet_CharityDetailsPortlet_priv_r_p_mvcRenderCommandName=%2Faccounts-and-annual-returns&_uk_gov_ccew_onereg_charitydetails_web_portlet_CharityDetailsPortlet_priv_r_p_organisationNumber=3956536

95. University of Oxford website [Internet]. Co-PACT summary. Available from: https://www.psych.ox.ac.uk/research/chimes/co-pact/co-pact_summary

96. ImROC [Internet]. n.d. Available from: https://imroc.org/resource/co-production-sharing-our-experiences-reflecting-on-our-learning/

97. Killaspy H, Rambarran D, Harden C, Fearon D, Caren G, McClinton K. A comparison of service users placed out of their local area and local rehabilitation service users. Journal of Mental Health. 2009 Jan;18(2):111–20.

98. Bhui KS, Owiti JA, Palinski A, Ascoli M, De Jongh B, Archer J, et al. A cultural consultation service in East London: Experiences and outcomes from implementation of an innovative service. International Review of Psychiatry. 2015 Jan 2;27(1):11–22.

99. Killaspy H. Supported accommodation for people with mental health problems. World Psychiatry. 2016 Feb;15(1):74–5.

100. National Guideline Alliance. Rehabilitation in adults with complex psychosis and related severe mental health conditions [A] Identifying people who would benefit most from mental health rehabilitation services [NICE Guideline NG181 Evidence Review] [Internet]. The National Institute for Health and Care Excellence; 2020. Available from: https://www.nice.org.uk/guidance/ng181/evidence/a-identifying-people-who-would-benefit-most-from-mental-health-rehabilitation-services-pdf-8833147166

101. National Guideline Alliance. Rehabilitation in adults with complex psychosis and related severe mental health conditions [P] The features of supported accommodation and housing that promote successful community living [NICE Guideline No. 181 Evidence Review] [Internet]. The National Institute for Health and Care Excellence; 2020. Available from: https://www.nice.org.uk/guidance/ng181/evidence/p-the-features-of-supported-accommodation-and-housing-that-promote-successful-community-living-pdf-317993297947

102. Dalton-Locke C, Marston L, McPherson P, Killaspy H. The Effectiveness of Mental Health Rehabilitation Services: A Systematic Review and Narrative Synthesis. Front Psychiatry. 2021 Jan 13;11:607933.

103. Homeless Link. Housing first in England: The principles. [Internet]. 2016. Available from: https://www.dahalliance.org.uk/media/10827/1-the-principles-for-housing-first.pdf

104. Gilmer TP, Stefancic A, Ettner SL, Manning WG, Tsemberis S. Effect of Full-Service Partnerships on Homelessness, Use and Costs of Mental Health Services, and Quality of Life Among Adults With Serious Mental Illness. Arch Gen Psychiatry. 2010 Jun 1;67(6):645.

105. Gilmer TP, Stefancic A, Tsemberis S, Ettner SL. Full-Service Partnerships Among Adults With Serious Mental Illness in California: Impact on Utilization and Costs. PS. 2014 Sep;65(9):1120–5.

106. van Bilsen HPJG. Lessons to be learned from the oldest community psychiatric service in the world: Geel in Belgium. BJPsych Bull. 2016 Aug;40(4):207–11.

107. National Empowerment Center. power2u. 2023. Healing Homes: An Alternative, Swedish Model for Healing Psychosis. Available from: https://power2u.org/evidence-for-peer-run-crisis-alternatives/

108. Macpherson R, Edwards TR, Chilvers R, David C, Elliott HJ. Twenty-four hour care for schizophrenia. Cochrane Schizophrenia Group, editor. Cochrane Database of Systematic Reviews [Internet]. 2009 Apr 15 [cited 2023 Jun 23]; Available from: https://doi.wiley.com/10.1002/14651858.CD004409.pub2

109. Maguire T, Ryan J, Fullam R, McKenna B. Evaluating the Introduction of the Safewards Model to a Medium- to Long-Term Forensic Mental Health Ward. Journal of Forensic Nursing. 2018 Oct;14(4):214–22.

110. Casadio R, Marin IC, Thomé T, Mezzina R, Baker P, Jenkins J, et al. The Recovery House in Trieste: rational, participants, intervention as the “work.” TC. 2018 Dec 10;39(4):149–61.

111. NHS England. The offender personality disorder pathway strategy. National Offender Management Service. [Internet]. 2015. Available from: https://www.england.nhs.uk/commissioning/wp-content/uploads/sites/12/2016/02/opd-strategy-nov-15.pdf

112. Social Interest Group. Penrose OPD HASS [Internet]. Available from: https://socialinterestgroup.org.uk/our-services/penrose-opd-hass-catford/

113. Social Interest Group. OPD HASS Male Housing & Accommodation Support Service [Internet]. Available from: https://socialinterestgroup.org.uk/wp-content/uploads/2020/05/OPD-HASS-Leaflet-new.pdf

114. Catanesi R, Mandarelli G, Ferracuti S, Valerio A, Carabellese F. THE NEW ITALIAN RESIDENTIAL FORENSIC PSYCHIATRIC SYSTEM (REMS). A ONE-YEAR POPULATION STUDY. ITALIAN JOURNAL OF CRIMINOLOGY. 2020 Mar 30;13(V. 13 (2019): Rassegna Italiana di Criminologia-Numero Speciale):7–23.

115. Think Local Act Personal [Internet]. n.d. Shared Lives Plus Community alternatives to residential care. Available from: https://www.thinklocalactpersonal.org.uk/innovations-in-community-centred-support/directory/Community-alternative-to-residential-care/#:~:text=Shared%20Lives%20is%20used%20by,who%20misuse%20substances%2C%20and%20offenders.

116. Substance Abuse and Mental Health Services Administration. Forensic Assertive Community Treatment (FACT): A Service Delivery Model for Individuals with Serious Mental Illness Involved With the Criminal Justice System [Internet]. Substance Abuse and Mental Health Services Administration (SAMHSA); n.d. Available from: https://store.samhsa.gov/sites/default/files/d7/priv/pep19-fact-br.pdf

117. Lamberti JS, Weisman RL. Essential Elements of Forensic Assertive Community Treatment. Harv Rev Psychiatry. 2021 Jul;29(4):278–97.

118. Craissati J, Ramsden J, Ryan S, Webster N, West L. Intensive intervention and risk management services (IIRMS) three years on: what we need to do better in the offender personality disorder pathway. JFP. 2021 Dec 1;23(4):385–96.

119. O’Meara A, Morgan L, Godden S, Davies J. A model of a specialist transitional support and liaison service within the Offender Personality Disorder Pathway in Wales: Learning from a regional pilot service. The Journal of Community and Criminal Justice. 2019;

120. Ching H, Daffern M, Martin T, Thomas S. Reducing the use of seclusion in a forensic psychiatric hospital: assessing the impact on aggression, therapeutic climate and staff confidence. Journal of Forensic Psychiatry & Psychology. 2010 Oct;21(5):737–60.

121. Maguire T, Young R, Martin T. Seclusion reduction in a forensic mental health setting: Seclusion reduction in a forensic setting. Journal of Psychiatric and Mental Health Nursing. 2012 Mar;19(2):97–106.

122. Maguire T, Ryan J, Fullam R, McKenna B. Safewards Secure: A Delphi study to develop an addition to the Safewards model for forensic mental health services. Psychiatric Ment Health Nurs. 2022 Jun;29(3):418–29.

123. de Boer J, Gerrits J. Learning from Holland: the TBS system. Psychiatry. 2007 Nov;6(11):459–61.

124. Senn D, Bulten E, Tomlin J, Völlm B. A Comparison of English and Dutch Long-Stay Patients in Forensic Psychiatric Care. Front Psychiatry. 2020 Nov 30;11:574247.

125. Kuester L, Freestone M, Seewald K, Rathbone R, Bhui K. Evaluation of Psychologically Informed Planned Environments (PIPEs) Assessing the first five years [Internet]. HM Prison & Probation Service; 2022. Available from: https://www.gov.uk/government/publications/evaluation-of-psychologically-informed-planned-environments

126. Midlands Partnership University NHS Trust. Forensic Mental Health: Offender Personality Disorder Pathways [Internet]. Available from: https://forensics.mpft.nhs.uk/offender-personality-disorder-pathways

127. Jarvis D, Shaw J, Lovell T. Service user experiences of a psychologically enhanced resettlement service [PERS] in an English open prison. JFP. 2022 Jun 28;24(3):241–52.

128. Lamb CE. Alternatives to admission for children and adolescents: providing intensive mental healthcare services at home and in communities: what works? Current Opinion in Psychiatry. 2009 Jul;22(4):345–50.

129. McDougall T, Worrall-Davies A, Hewson L, Richardson G, Cotgrove A. Tier 4 Child and Adolescent Mental Health Services (CAMHS) - Inpatient Care, Day Services and Alternatives: An Overview of Tier 4 CAMHS Provision in the UK. Child and Adolescent Mental Health. 2008 Nov;13(4):173–80.

130. Fisher PA, Gilliam KS. Multidimensional Treatment Foster Care: An Alternative to Residential Treatment for High Risk Children and Adolescents. Psychosocial Intervention. 2012 Aug;21(2):195–203.

131. Hart D, La Velle I. Secure children’s homes: placing welfare and justice children together. [Internet]. 2021. Available from: https://assets.publishing.service.gov.uk/government/uploads/system/uploads/attachment_data/file/983619/Secure_children_s_homes_placement_review_report.pdf

132. Secure Children’s Homes. Secure Children’s Homes [Internet]. 2023. Available from: http://securechildrenshomes.org.uk

133. Family Rights Group. Helping Families Helping Children [Internet]. Available from: https://frg.org.uk

134. Grizenko N, Papineau D, Sayegh L. Effectiveness of a Multimodal Day Treatment Program for Children with Disruptive Behavior Problems. Journal of the American Academy of Child & Adolescent Psychiatry. 1993 Jan;32(1):127–34.

135. McCarthy G, Baker S, Betts K, Bernard D, Dove J, Elliot M, et al. The Development of a New Day Treatment Program for Older Children (8–11 Years) with Behavioural Problems: The Go Zone. Clin Child Psychol Psychiatry. 2006 Jan;11(1):156–66.

136. Muskens JB, Herpers PCM, Hilderink C, van Deurzen PAM, Buitelaar JK, Staal WG. Intensive home treatment for adolescents in psychiatric crisis. BMC Psychiatry. 2019 Dec;19(1):412.

137. Evans ME, Boothroyd RA, Armstrong MI, Greenbaum PE, Brown EC, Kuppinger AD. An Experimental Study of the Effectiveness of Intensive In-Home Crisis Services for Children and Their Families: Program Outcomes. Journal of Emotional and Behavioral Disorders. 2003 Apr;11(2):92–102.

138. Northover G. Children and Young People’s Mental Health Services GIRFT Programme National Specialty Report [Internet]. 2022. Available from: https://future.nhs.uk/connect.ti/GIRFTNational/view?objectId=130556421

139. NHS North Central London Integrated Care Board. Child & Adolescent Mental Health Service [Internet]. n.d. Available from: https://gps.northcentrallondon.icb.nhs.uk/services/child-and-adolescent-mental-health-service-camhs-islington

140. Boege I, Corpus N, Schepker R, Kilian R, Fegert JM. Cost-effectiveness of intensive home treatment enhanced by inpatient treatment elements in child and adolescent psychiatry in Germany: A randomised trial. Eur psychiatr. 2015 Jul;30(5):583–9.

141. Ougrin D, Zundel T, Corrigall R, Padmore J, Loh C. Innovations in Practice: pilot evaluation of the supported discharge service (SDS): clinical outcomes and service use. Child Adolesc Ment Health. 2014 Nov;19(4):265–9.

142. Ougrin D, Corrigall R, Stahl D, Poole J, Zundel T, Wait M, et al. Supported discharge service versus inpatient care evaluation (SITE): a randomised controlled trial comparing effectiveness of an intensive community care service versus inpatient treatment as usual for adolescents with severe psychiatric disorders: self-harm, functional impairment, and educational and clinical outcomes. Eur Child Adolesc Psychiatry. 2021 Sep;30(9):1427–36.

143. Ougrin D, Corrigall R, Poole J, Zundel T, Sarhane M, Slater V, et al. Comparison of effectiveness and cost-effectiveness of an intensive community supported discharge service versus treatment as usual for adolescents with psychiatric emergencies: a randomised controlled trial. The Lancet Psychiatry. 2018 Jun;5(6):477–85.

144. Seattle Children’s Hospital Research Foundation. Behavioral Health Crisis Care Clinic [Internet]. 2023. Available from: https://www.seattlechildrens.org/clinics/psychiatry-and-behavioral-medicine/services/behavioral-health-crisis-care-clinic/

145. Evans ME, Boothroyd RA, Armstrong MI. Development and Implementation of an Experimental Study of the Effectiveness of Intensive In-Home Crisis Services for Children and Their Families. Journal of Emotional and Behavioral Disorders. 1997 Apr;5(2):93–105.

146. Broersen M, Creemers DHM, Frieswijk N, Vermulst AA, Kroon H. Effects of Youth Flexible Assertive Community Treatment: outcomes of an 18-month observational study. Soc Psychiatry Psychiatr Epidemiol [Internet]. 2023 Jun 6 [cited 2023 Aug 9]; Available from: https://link.springer.com/10.1007/s00127-023-02508-x

147. Broersen M, Frieswijk N, Coolen R, Creemers DHM, Kroon H. Case Study in Youth Flexible Assertive Community Treatment: An Illustration of the Need for Integrated Care. Front Psychiatry. 2022 May 12;13:903523.

148. Baugh C, Blanchard E, Hopkins I, editors. Quality Standards for Liaison Psychiatry Services, Sixth Edition [Internet]. Psychiatric Liaison Accreditation Network (PLAN) & Royal College of Psychiatrists; 2020. Available from: https://www.rcpsych.ac.uk/docs/default-source/improving-care/ccqi/quality-networks/psychiatric-liaison-services-plan/quality-standards-for-liaison-psychiatry-services---sixth-edition-20209b6be47cb0f249f697850e1222d6b6e1.pdf?sfvrsn=1ddd53f2_0

149. CQC. Review of children and young people’s mental health services. Phase One supporting documentation: Summary of recent policy and literature [Internet]. Care Quality Commission; 2017. Available from: https://www.cqc.org.uk/sites/default/files/20171027_cypmhphase1_literaturereview.pdf

150. Central & North West London NHS Foundation Trust. Community Eating Disorder Service for Children and Young People [Internet]. Available from: https://www.cnwl.nhs.uk/services/mental-health-services/eating-disorders/community-eating-disorder-service-children-and-young-people

151. SAMHSA. Substance Abuse and Mental Health Services Administration: National Guidelines for Child and Youth Behavioral Health Crisis Care [Internet]. Substance Abuse and Mental Health Services Administration; 2022. Available from: https://store.samhsa.gov/sites/default/files/SAMHSA_Digital_Download/pep-22-01-02-001.pdf

152. Saxon V, Mukherjee D, Thomas D. Behavioral Health Crisis Stabilization Centers: A New Normal. J Mental Health & Clin Psychology. 2018 May 1;2(3):23–6.

153. Hamilton B, Fletcher J, Sands N, Roper C, Elsom S. Safewards Victorian Trial Final Evaluation Report [Internet]. The University of Melbourne; 2016. Available from: https://www.health.vic.gov.au/sites/default/files/migrated/files/collections/research-and-reports/s/safewards-final-evaluation-report.pdf

154. Hottinen A, Rytilä‐Manninen M, Laurén J, Autio S, Laiho T, Lindberg N. Impact of the implementation of the safewards model on the social climate on adolescent psychiatric wards. Int J Mental Health Nurs. 2020 Jun;29(3):399–405.

155. Westling S. Brief Admission for Adolescents Who Self-Harm ClinicalTrials.gov Protocol [Internet]. 2022. Available from: https://classic.clinicaltrials.gov/ct2/show/NCT04962373

156. South West London and St George’s Mental Health NHS Trust [Internet]. n.d. Corner House - National Deaf CAMHS. Available from: https://www.swlstg.nhs.uk/our-services/find-a-service/service/corner-house-national-deaf-camhs#:~:text=Corner%20House%20is%20a%20six,complex%20emotional%20and%20psychological%20problems.

157. Central and North West London NHS Foundation Trust. Collingham Inpatient Service [Internet]. 2023. Available from: https://www.cnwl.nhs.uk/camhs/our-services/inpatient/collingham-inpatient-service-2
